# Chalcone Derivatives: Role in Anticancer Therapy

**DOI:** 10.3390/biom11060894

**Published:** 2021-06-16

**Authors:** Yang Ouyang, Juanjuan Li, Xinyue Chen, Xiaoyu Fu, Si Sun, Qi Wu

**Affiliations:** 1 Department of Breast and Thyroid Surgery, Renmin Hospital of Wuhan University, Wuhan 430060, China; 2014302180180@whu.edu.cn (Y.O.); snowy1150219@whu.edu.cn (J.L.); chenxinyue@whu.edu.cn (X.C.); 2016302180206@whu.edu.cn (X.F.); 2 Department of Clinical Laboratory, Renmin Hospital of Wuhan University, Wuhan 430060, China

**Keywords:** chalcone, anticancer, molecular targets, bioactive dietary compounds

## Abstract

Chalcones (1,3-diaryl-2-propen-1-ones) are precursors for flavonoids and isoflavonoids, which are common simple chemical scaffolds found in many naturally occurring compounds. Many chalcone derivatives were also prepared due to their convenient synthesis. Chalcones as weandhetic analogues have attracted much interest due to their broad biological activities with clinical potentials against various diseases, particularly for antitumor activity. The chalcone family has demonstrated potential in vitro and in vivo activity against cancers via multiple mechanisms, including cell cycle disruption, autophagy regulation, apoptosis induction, and immunomodulatory and inflammatory mediators. It represents a promising strategy to develop chalcones as novel anticancer agents. In addition, the combination of chalcones and other therapies is expected to be an effective way to improve anticancer therapeutic efficacy. However, despite the encouraging results for their response to cancers observed in clinical studies, a full description of toxicity is required for their clinical use as safe drugs for the treatment of cancer. In this review, we will summarize the recent advances of the chalcone family as potential anticancer agents and the mechanisms of action. Besides, future applications and scope of the chalcone family toward the treatment and prevention of cancer are brought out.

## 1. Introduction

Cancer is caused by the uncontrolled growth of cells and is a multifactorial disease that claims millions of lives each year worldwide. Its genesis and progression are extremely complex. A variety of strategies are applied to anticancer treatments, including surgery, chemotherapy, and radiotherapy used alone or in combination. However, multidrug resistance (MDR) and side effects constitute major impediments to effective cancer therapy [1]. Phytochemicals, such as chalcones, have been shown to be inexpensive, readily available and relatively nontoxic. Certain chalcones can target key molecular reactions that may induce the genesis and progression of cancer [2]. Thus, scientists are using traditional knowledge of medicinal plants and the sustainable exploitation of marine natural products to synthesize new, more powerful and effective therapeutic antitumor drugs by leveraging different molecular mechanisms [3,4,5].

**Figure 1:**
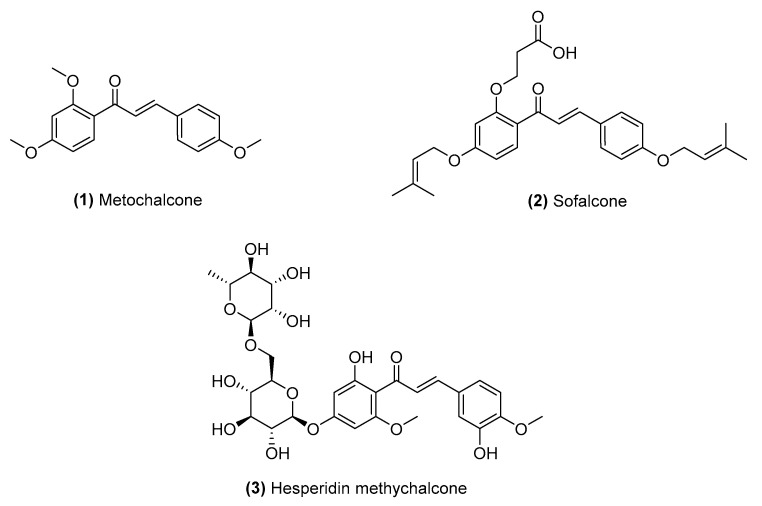
Chemical structures of approved and clinically tested chalcones.

A chalcone is a simple chemical scaffold in many natural plant products, including spices, vegetables, fruits, teas [6,7,8,9]. Chalcones, which belong to the flavonoid family and act as intermediates in the biosynthesis of flavonoids, exhibit structural heterogeneity and can act on various drug targets. Chalcone family members have received considerable attention not only because of the possibilities for their synthetic and biosynthetic production but also because of the scope of their biological activities, including anticancer [10], anti-inflammatory [11], antidiabetic [12], cancer chemopreventive [13], antioxidant [14], antimicrobial [15], antileishmanial [16] and antimalarial activities [17]. More importantly, several chalcone compounds have been approved for market and clinical use for various health conditions [e.g., as metochalcone-choleretic/diuretics (**1**); sofalcone-based anti-ulcer/mucoprotectives (**2**); and hesperidin methylchalcone-vascular protectives (**3**)], exemplifying the clinical potential of chalcones [2,8,9,18] (Figure 1).

Chalcone compounds have a chemical scaffold of 1,3-diaryl-2-propen-1-one, which can be conveniently modified to alter the biological activities of these molecules. By adding various functional groups (aryls, halogens, hydroxyls, carboxyls, phenyl, etc.) [18], which enable chalcone binding with different molecular targets and, as compounds, interaction with other molecules, chalcones exhibit a broad spectrum of biological activities. Therefore, chalcones are useful templates for the development of novel anticancer agents. Moreover, hybridization of the chalcone moiety with other anticancer pharmacophores produces hybrids that have the potential to overcome drug resistance and improve therapeutic specificity, rendering it a promising strategy for developing novel anticancer agents. In this review, we focus on the medicinal chemistry strategies employed for the design and development of anticancer chalcones. The multiple mechanisms of anticancer activities exhibited by chalcones and their therapeutic potential are also summarized herein.

## 2. Strategies Employed to Produce Anticancer Chalcones

**Figure 2:**
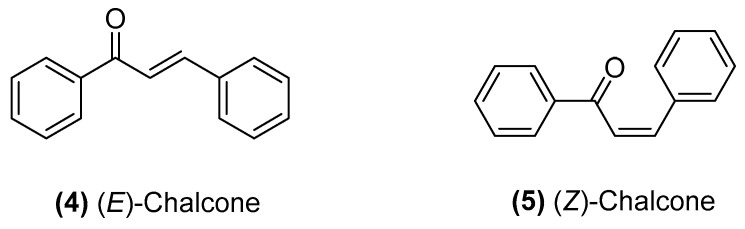
Structural of chalcone scaffold.

**Scheme 1:**
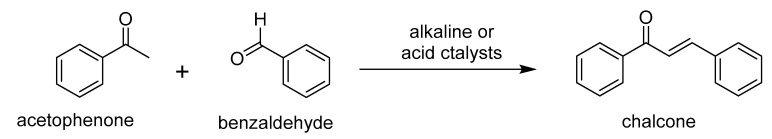
Claisen–Schmidt condensation of chalcone.

**Figure 3:**
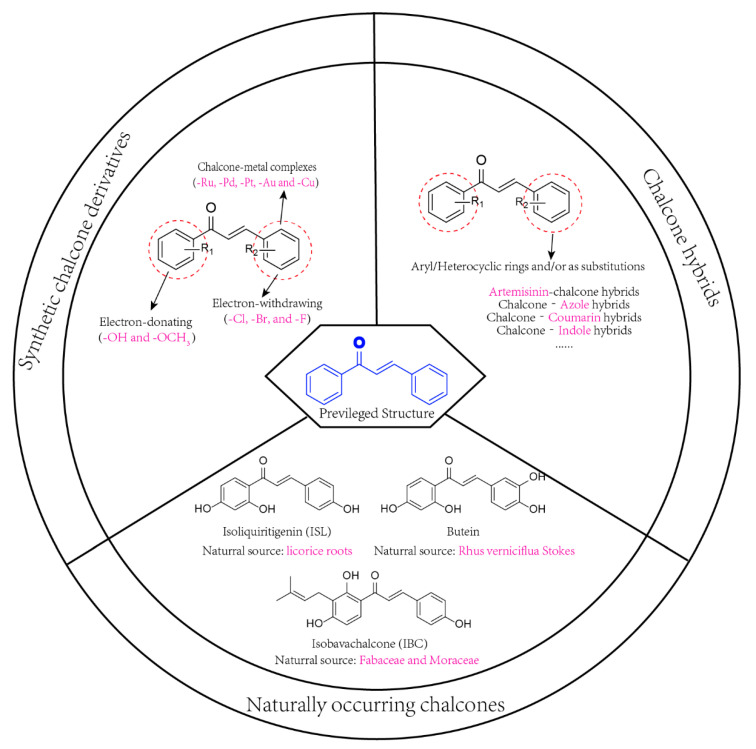
Strategies Employed to Produce Anticancer Chalcones.

Chalcone compounds have a chemical scaffold of 1,3-diaryl-2-propen-1-one in trans- (**4**) or cis- (**5**) isomers with two aromatic rings (rings A and B) that are joined by a three-carbon unsaturated α,β-carbonyl system (Figure 2). In most cases, the trans isomer is thermodynamically more stable, and therefore, it is the predominant configuration among chalcones [19]. In addition, chalcones contain many replaceable hydrogens, which enables the use of various methods and schemes for the synthesis of chalcone derivatives [20]. In each of these methods, the most important part is the condensation of two aromatic systems (with nucleophilic and electrophilic groups) to yield the chalcone scaffold. The reaction scheme used in the synthesis of the standard scaffold of chalcones (1,3-diphenyl-2-propen-1-one) includes Claisen–Schmidt condensation, carbonylative Heck coupling reaction, coupling reaction, Sonogashira isomerization coupling reaction, continuous-flow deuteraction reaction, Suzuki–Miyaura coupling reaction, one-pot synthesis and solid acid catalyst-mediated reaction [9,18]. Amongst all methods, the Claisen–Schmidt condensation (Scheme 1) is one of the most common. Thus, the chalcone moiety is a useful template for the development of novel anticancer agents. In addition to the potent anticancer activity of naturally occurring chalcones, three-pronged strategies are employed for the synthesis of anticancer chalcone: structural manipulation of two aryl rings, the substitution of aryl rings to generate heteroaryl scaffolds, and/or molecular hybridization through conjugation with other pharmacologically interesting scaffolds to enhance the molecular anticancer properties [6,10] (Figure 3).

### 2.1. Naturally Occurring Chalcones

**Figure 4:**
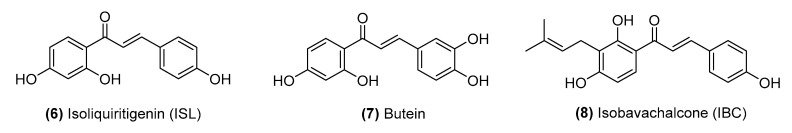
Structures of the naturally derived chalcones.

Chalcones constitute the core of many natural biological compounds and have been extensively studied for decades. The chalcone family has a wide range of structural diversity and can be roughly classified into two categories: simple/classical chalcones and hybrid chalcones with the core 1,3-diaryl-2-propen-1-one scaffold. They are widely distributed in various parts (roots, rhizomes, heartwood, buds, leaves, flowers, and seeds) of species of genera *Angelica*, *Sophora*, *Glycyrrhiza*, *Humulus*, *Scutellaria*, *Parartocarpus*, *Ficus*, *Dorstenia*, *Morus*, *Artocarpus*, and so forth [21,22]. Most natural chalcones occur in monomeric form. Their great structural diversity stems from the number and position of various substituents. Some prominent examples of this class of anticancer chalcones have been isolated from natural sources, such as isoliquiritigenin, butein, and isobavachalcone, and their potential anticancer activities are described herein (Figure 4).

Isoliquiritigenin (**6**) (2′,4′,4-trihydroxychalcone, ISL) is one of the most important bioactive compounds with a chalcone structure isolated from licorice roots. ISL is known to have therapeutic potential against various cancers, including breast cancer, colon cancer, gastrointestinal cancer, lung cancer, ovarian cancer, leukemia, and melanoma [23]. Furthermore, ISL not only inhibits cancer cell migration and invasion by suppressing cell proliferation [24], inducing apoptosis and autophagy [25,26], arresting the cell cycle [27], inhibiting angiogenesis [28] and obstructing metastasis [29], but can also enhance chemosensitivity [30].

Butein (**7**), a biologically active flavonoid, is derived from the bark of *Rhus verniciflua* Stokes and exhibits significant anticancer activity in many types of cancers [31,32]. Butein shows anticarcinogenic action in non-small cell lung cancer (NSCLC) through endoplasmic reticulum stress-dependent Reactive Oxygen Species (ROS) generation and an apoptosis pathway both in vivo and in vitro [33]. In addition, butein induces G2/M phase cell cycle arrest by inhibiting Aurora B and histone H3 phosphorylation in hepatocellular carcinoma (HCC) [34]. Butein is also involved in G2/M phase arrest and apoptosis by increasing the phosphorylation of ataxia telangiectasia mutated (ATM) and checkpoint kinase-1 and 2 (Chk1/2), thereby reducing cell division cycle 25C (cdc25C) levels in HCC [35]. Moreover, butein can attenuate angiogenesis [36], cell invasion/metastasis, and inflammation [37,38] by inhibiting the nuclear factor kappa-light-chain-enhancer of activated B cells (NF-κB) signal pathway [39].

Isobavachalcone (**8**) (IBC) is one of the most useful active compounds among numerous chalcones, and it is predominantly found in plants of the Fabaceae and Moraceae families [40]. IBC exhibits antitumor activity against numerous cancer types. IBC has been found to exhibit antiproliferative and proapoptotic activities in HCC by targeting the extracellular signal-regulated kinases (ERKs)/ribosomal S6 kinase 2 (RSK2) signaling pathway [41]. Moreover, IBC has been found to induce ROS-mediated apoptosis by targeting thioredoxin reductase 1 (TrxR1) in human prostate cancer [42]. IBC has also been demonstrated to inhibit cell proliferation and induce apoptosis by suppressing the AKT/glycogen synthase kinase 3β (GSK3β)/β-catenin pathway in colorectal cancer cells [43]. Additionally, IBC can inhibit estrogen receptor alpha (ERα) and decrease CD44 antigen expression, which leads to decreased paclitaxel resistance in ER+ breast cancer [44]. Studies have also reported that IBC inhibits tumor formation in mouse skin cancer [45] and induces apoptosis in neuroblastoma [46].

### 2.2. Synthetic Chalcone Derivatives

**Figure 5:**
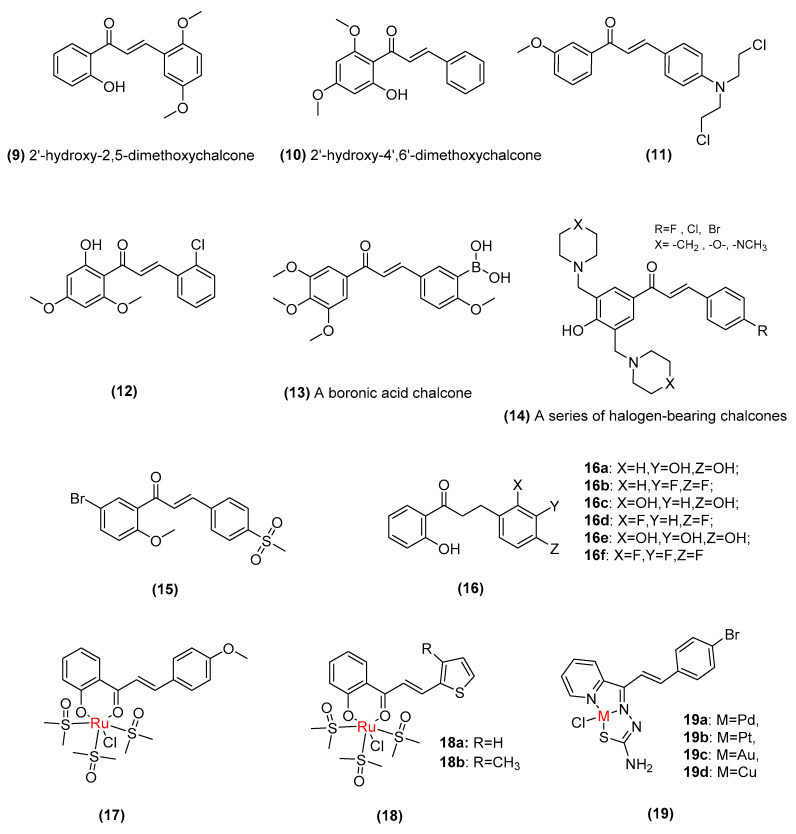
Structure of synthetically derived chalcone.

The successful and significant use of naturally occurring chalcones as potential anticancer agents has inspired many efforts directed to developing novel synthetic chalcones with anticancer properties. Furthermore, investigations into the molecular modification of the chalcone structure have promoted chemical alterations, which lead to improved chalcone physicochemical properties and biological profiles. Among the molecular modification strategies used, structural manipulation of both aryl rings, replacement of aryl rings with heteroaryl/alicyclic/steroidal scaffolds and molecular hybridization are the most widely used examples [6]. The standard scaffold in chalcones (1,3-diphenyl-2-propen-1-one) is the most widely researched chalcone structure for its potential anticancer activity. Structural manipulations of 1,3-diphenyl-2-propen-1-one are mostly focused on the phenyl rings (A and B). We summarize the effects of structure–activity relationship (SAR) substitution patterns on chalcone anticancer properties in the next section, including electron-donating (-OH and -OCH_3_), electron-withdrawing (-Cl, -Br, and -F) and chalcone–metal complexes (Figure 5).

Similar to naturally occurring chalcones, hydroxyl and methoxy groups added to specific positions on the phenyl ring contribute to the anticancer activity of synthetic compounds. A recent study found that 2′-hydroxy-2,5-dimethoxychalcone (**9**, half-maximal inhibitory concentration [IC_50_]: 9.76-40.83 µM) and 2′-hydroxy-4′,6′-dimethoxychalcone (**10**, IC_50_: 9.18–46.11 µM) exhibit antiproliferative and proapoptotic activity in a panel of canine lymphoma and leukemia cell lines [47]. Elkhalifa et al. reported that ((*E*)-3-(4-(Bis(2-chloroethyl)amino)phenyl)-1-(3-methoxyphenyl)prop-2-en-1-one) (**11**, IC_50_: 3.94–9.22 µM) significantly inhibit tumor invasion and migration in triple-negative breast cancer (TNBC) by inducing cell cycle arrest and promoting apoptosis [48]. Moreover, Kachadourian et al. demonstrated that (*E*)-3-(2-chlorophenyl)-1-(2-hydroxy-4,6-dimethoxyphenyl)prop-2-en-1-one (**12**), an alternate structure of 30 synthetic chalcone derivatives, can induce NF-E2-related factor 2 (Nrf2) transcriptional activity and increase intracellular levels of glutathione (GSH) [49]. Kong et al. sought to determine whether methoxylated chalcone analogs of combretastatin A-4, the boronic acid chalcone (**13**, the concentration that causes 50% growth inhibition [GI_5__0_]: 10–200 nm), which involve reversible, high-affinity binding at the colchicine site of tubulin, show prominent anticancer activity in a variety of cancer cell lines [50,51]. These methoxylated chalcone studies indicated that the number and position of methoxy substituents on the aromatic rings seem to play important roles in cytotoxicity.

A series of halogen-bearing chalcones (**14**, the median cytotoxic concentration [CC_50_]: 1.6–18.4 µM) that combine a basic chalcone framework with a halogen moiety (F, Cl, Br, etc.) have been found to exhibit significant, remarkable cytotoxic potencies and selective toxicity for tumors [52]. Zhang et al. reported a novel brominated chalcone derivative (**15**, IC_50_: 3.57–5.61 µM) with antiproliferative activity in gastric cancer cells in vitro and in vivo involving ROS-mediated upregulation of death receptors death receptors 5 (DR5) and DR4 expression and apoptosis [53]. Moreover, Padhye et al. found that fluorinated chalcones (**16**, IC_50_: 18.67 µM and 26.43 µM) show more potent antioxidant and antiproliferative activity against human pancreatic BxPC-3 cancer cells and human breast cancer BT-20 cells than their hydroxyl counterparts [54].

In recent years, chalcone–metal complexes have attracted widespread attention in bioinorganic medicinal chemistry due to their chelation/coordination properties with various metals and their regulatory effects on various anticancer targets [55]. A recent study found that new Ru(II)-DMSO (**17**, IC_50_: 15–28.64 µM) complexes with substituted chalcone ligands show significant anti-breast-cancer activity by inhibiting DNA topoisomerase [56]. Similarly, Jovanovic et al. reported that two thiophene-substituted chalcone–ruthenium complexes with the general formula cis-[Ru(S-DMSO)3(R-CO-CH=CH-R’)Cl] (**18**, IC_50_: 22.9–76.8 µM) act as anticancer agents in HeLa cells (human cervical cancer cells) with cytotoxic and proapoptotic activity that can remarkably inhibit topoisomerase II and strongly bind with DNA [57]. Moreover, Samia et al. synthesized palladium (**19a**), platinum (**19b**), gold (**19c**), and copper (**19d**) complexes with (*E*)-3-(4-bromophenyl)-1-(pyridin-2-yl)prop-2-en-1-one thiosemicarbazone (HPyCT4BrPh) and explored the cytotoxic potential against HL-60 (human promyelocytic leukemia), THP-1 (human monocytic leukemia) cells, MDAMB-231 (human metastatic breast cancer cell line) and MCF-7 (Michigan Cancer Foundation-7 human breast adenocarcinoma) cell lines (IC_50_: 0.16–1.27 µM). The coordination of HPyCT4BrPh complexes to copper and gold has been proven to lead to high antitumor activity [58,59].

### 2.3. Chalcone Hybrids

Hybrid molecules have the potential not only to overcome drug resistance but also to exhibit increased activity and enhanced specificity [60,61,62]. Therefore, hybridization of the chalcone moiety with other anticancer pharmacophores is a promising method for developing novel anticancer agents [10]. Recently, a number of chalcone hybrids have been prepared and evaluated for their anticancer activity; some were found to have remarkable activity both in vitro and in vivo, exhibiting their potential as anticancer drugs.

#### 2.3.1. Artemisinin–Chalcone Hybrids

**Figure 6:**
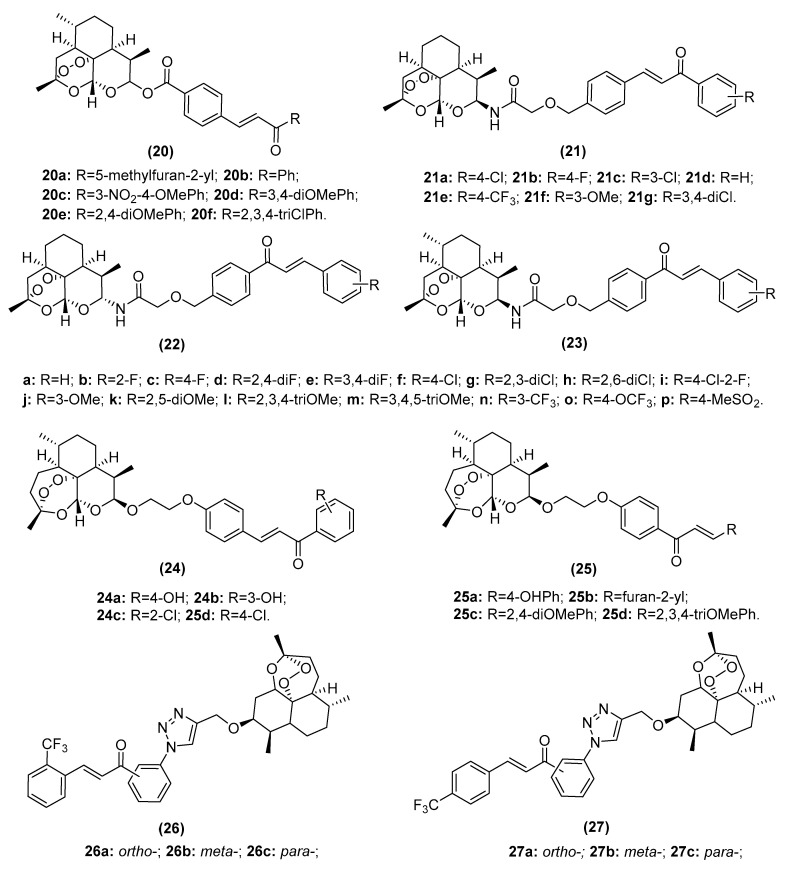
Chemical structures of artemisinin–chalcone hybrids.

Artemisinin derivatives bear a peroxide-containing sesquiterpene lactone moiety and can produce highly reactive free radicals (peroxyl free radicals and ROS) in the presence of ferrous ions (Fe[II]) [63,64]. As tumor cells contain a much higher level of Fe[II] ions than healthy tissue cells, artemisinin derivatives can induce the formation of peroxyl free radicals and ROS and oxidative stress, DNA damage and selective apoptosis of cancer cells [65]. Therefore, hybridization of artemisinin and chalcone may provide novel anticancer candidates that not only induce potent toxicity in cancer cells but also exhibit high safety levels in normal cells. The chemical structures of artemisinin–chalcone hybrids are shown in Figure 6. 

Smit et al. synthesized a series of artemisinlcone hybrids (**20**, IC_50_: 1.02–53.7 µM) and found not only a potent effect against intraerythrocytic *Plasmodium falciparum* parasites but also considerable activity against TK-10 (renal), UACC-62 (melanoma), and MCF-7 cancer cell lines [66]. α-Configurated artemisinin–chalcone hybrids (**22**, IC_50_: 0.10–29 µM) and β-configurated analogs (**21**, IC_50_: 1.7–27 µM; **23**, IC_50_: 0.09–23 µM) also showed greater antiproliferative and cytotoxic effects than dihydroartemisinin against HT-29 (human colon cancer cell), A549, MDA-MB-231, HeLa, and H460 (human lung cancer cell) cancer cell lines [67,68]. Similarly, a novel series of artemisinlcone hybrids (**24**, **25**) prepared by Gaur et al. showed potent activity against HL-60 (leukemia), Mia PaCa-2 (pancreatic cancer), PC-3 (prostate cancer), LS180 (colon cancer) and HEPG2 (hepatocellular carcinoma) cancer cell lines with a high selectivity index [69]. Kapkoti et al. designed 1,2,3-triazole-containing artemisinin–chalcone hybrids (**26**, IC_50_: 7.16–57.18 µM; **27**, IC_50_: 17.14–69.67 µM) displaying remarkable activity against K562 (human chronic myeloid leukemia cell), PC-3 (human prostate cancer cell), A431 (human skin squamous cell carcinoma), MDA-MB-231 (human metastatic breast cancer cell line), COLO-205 (human colon cancer cell), A549 (human lung cancer cell), and HEK-293 (human embryonic kidney cell) cancer cell lines with significant induction of ROS formation [70]. Moreover, a toxicity study on human erythrocytes revealed that these molecules are nontoxic (IC_50_: >100 μg/mL).

#### 2.3.2. Chalcone–Azole Hybrids

**Figure 7:**
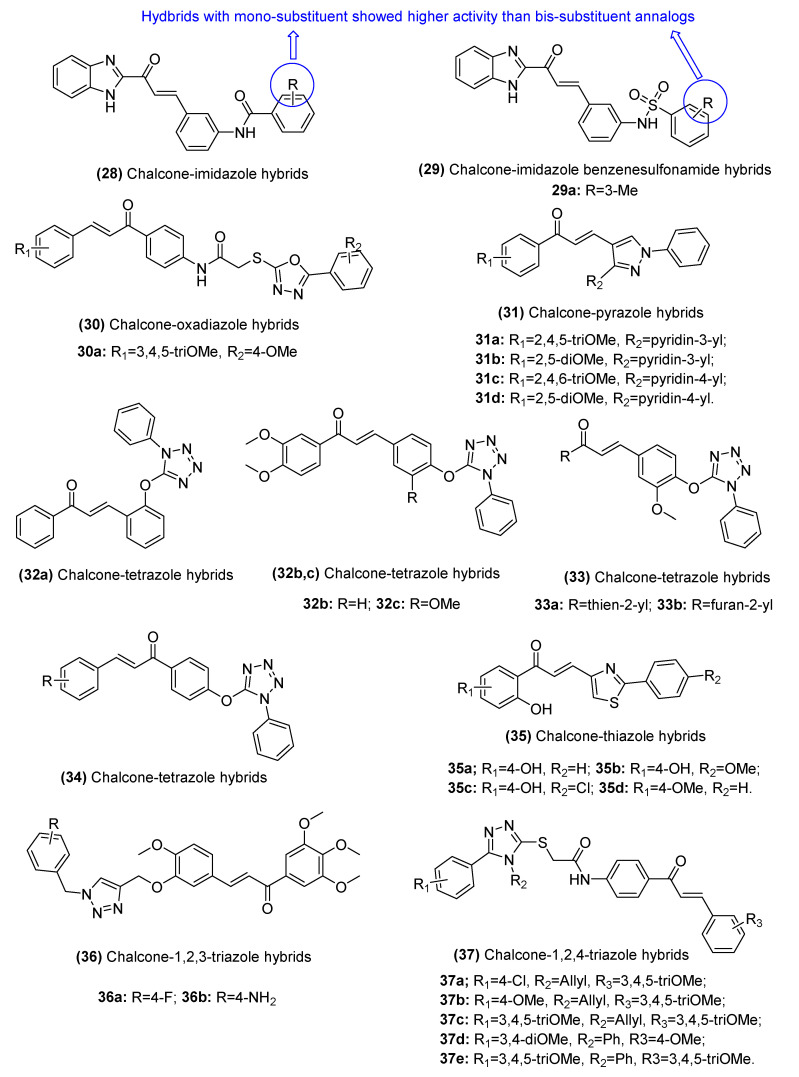
Chemical structures of chalcone–azole hybrids.

Azoles constitute a class of five-membered nitrogen-containing heterocyclic compounds with electron-rich properties [71] that includes imidazole, oxadiazole, pyrazole, tetrazole, thiazole, 1,2,3-triazole, and 1,2,4-triazole. Azoles are common pharmacophores used in the development of novel anticancer agents. Recently, a chalcone hybridized with an azole moiety showed significant potential as a novel anticancer agent (Figure 7). A series of chalcone–imidazole hybrids (**28**, IC_50_: 1.123–20.134 µM) bearing benzamide or benzenesulfonamide moieties (**29**, IC_50_: 0.597–19.995 µM) synthesized by Wang et al. display potential activity against HCT116, MCF-7, and 143B cancer cell lines in vitro. Molecular docking studies and enzymatic assays demonstrated that cathepsin (Cat) L and Cat K might be potential targets for these hybrids [72]. Chalcone–oxadiazole hybrids (**30**, GI_50_: 0.32–11.0 µM) have also shown broad spectrum activity against a series of 60 cancer cell lines, particularly leukemia cells. Furthermore, these synthesized compounds can inhibit epidermal growth factor receptor (EGFR) (IC_50_: 0.24 µM), proto-oncogene tyrosine-protein kinase (Src) (IC_50_: 0.96 µM), and interleukin-6 (IL-6) (% of control: 20%) and might play anticancer roles in various cancers [73]. 

Moreover, Hawash et al. reported that chalcone-pyrazole hybrids (**31**, IC_50_: 0.5–4.8 µM) cause cell cycle arrest in G2/M phase followed by apoptotic cell death and impaired cell growth in HCC cell lines [74], indicating that these hybrids may be considered potential chemotherapeutic agents for the treatment of HCC. A majority of chalcone–tetrazole hybrids (**32**, IC_50_: 0.6–3.7 μg/mL; **33**, IC_50_: 2.5–31.4 μg/mL; **34**, IC_50_: 12.0–42.4 μg/mL) have been shown to exert superior activity against colon HCT116, prostate PC-3, and breast MCF-7 cancer cell lines than cisplatin or fluorouracil [75].

Treatment with drug-resistant cell lines (CEM/ADR5000, MDA-MB-231/BCRP, HCT116(p53^−/−^), and U87MG/ΔEGFR cells) with thiazole-containing chalcone derivatives (**35**, IC_50_: 2.72–41.04 µM) was found to induce significant hypersensitivity effects, suggesting that these compounds are suitable molecules to combat the drug resistance of cancer cells [76]. Triazole has two structures: 1,2,3-triazole and 1,2,4-triazole. Both of these forms have shown potential as novel anticancer candidates. Both of them have shown their potential as novel anticancer candidates. Hussaini et al. synthesized a series of chalcone-1,2,3-triazole hybrids (**36**, GI_50_: 1.3–186.2 µM) with the same substituents as combretastatin-A4, which can lead to the accumulation of A549, HeLa, DU145, and HepG2 cancer cell lines in the G2/M phase, inhibit tubulin polymerization, and trigger apoptosis by inducing changes to the mitochondrial membrane potential and activating caspases 3 and 9 [77]. Moreover, Ahmed et al. prepared a series of novel 1,2,4-triazole/chalcone hybrids (**37**, IC_50_: 4.4–16.04 µM) that can induce the apoptosis of human lung adenocarcinoma A549 cells through a caspase-3-dependent pathway [78].

#### 2.3.3. Chalcone–Coumarin Hybrids

**Figure 8:**
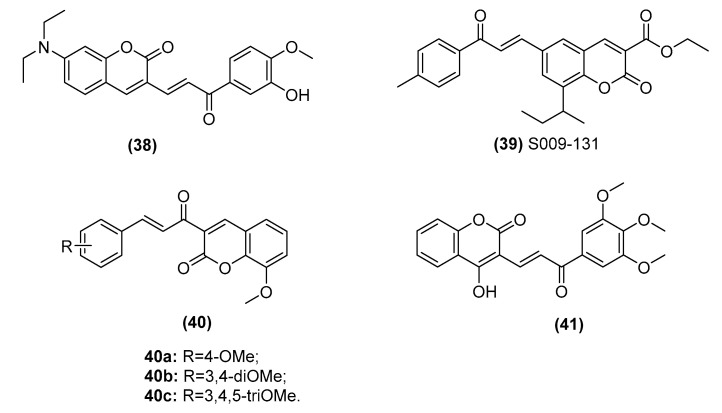
Chemical structures of chalcone–coumarin hybrids.

Coumarin derivatives have been shown to exhibit a variety of anticancer mechanisms, and some of these derivatives, such as irosustat, are in clinical trials for multiple cancer treatments, exhibiting their potential as putative anticancer drugs [79]. Thus, hybridization of chalcone with coumarin may produce attractive novel anticancer candidate agents (Figure 8). A recent report by Wang et al. demonstrated that a series of coumarlcone hybrids can decrease TrxR expression and significantly induce ROS accumulation to activate the mitochondrial apoptosis pathway. The representative hybrids (**38**, IC_50_: 3.6 μM) have exhibited higher antitumor activity against HCT116 cells than xanthohumol (Xn) [80]. Sinha et al. also reported chalcone–coumarin hybrids (**39**, S009-131) that show potential antiproliferation activity against cervical cancer cells (HeLa (IC_50_: 4.7 μM) and C33A (IC_50_: 7.6 μM)) by inducing apoptosis and arresting the cell cycle at G2/M phase [81,82,83]. This molecule induces an increase in the Bax/Bcl-2 ratio and intracellular ROS and then releases cytochrome c into the cytosol to activate the initiator caspase-9 and executioner caspase-3/7. Moreover, the tumor suppressor protein p53 and its transcriptional target p53 upregulated modulator of apoptosis (PUMA) are upregulated, suggesting their role in mediating cell death. In addition, chalcone–coumarin hybrids (**40**, IC_50_: 0.65–2.02 μM) have been shown to exert significant cytotoxic activity against HEPG2 liver cancer and K562 leukemia cells. Interestingly, these hybrids can also induce apoptosis by activating caspases 3 and 9 [84]. In addition, chalcone–coumarin hybrids (**41**, GI_50_: 22.11–41.08 μM) have been found to have antiproliferative activity in breast cancer cell lines (MDA-MB231, MDA-MB468, and MCF7 cells), which is comparable to cisplatin (GI_50_: 23.65–31.02 μM) [85,86].

#### 2.3.4. Chalcone–Indole Hybrids

**Figure 9:**
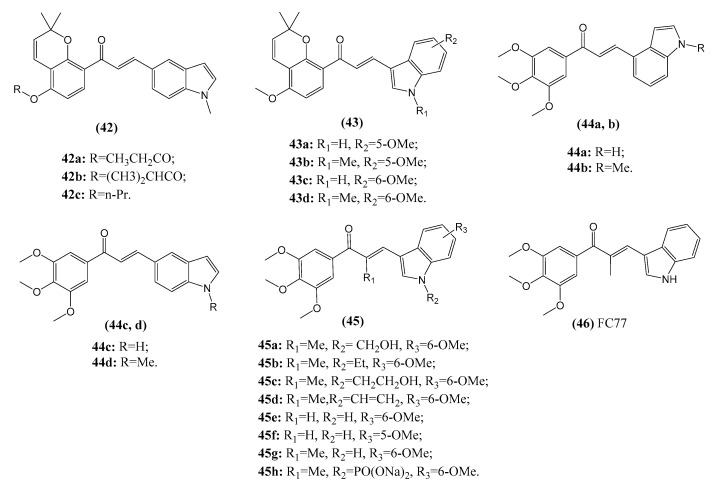
Chemical structures of chalcone–indole hybrids.

Due to the versatile nature of indoles, a variety of indole derivatives have been recently designed as anticancer agents that act via different mechanisms of action as histone deacetylases (HDACs), sirtuins, and DNA topoisomerase, etc. [87,88]. Several indole-containing drugs, such as semaxanib and sunitinib, have been used in the clinic for cancer treatment [89,90], and chalcone–ndole hybrids may represent a promising strategy to produce novel anticancer candidates (Figure 9). Chalcone–indole hybrids (**42**, IC_50_: 0.23–1.8 μM) synthesized by Wang et al. have been shown to display potent cytotoxic activity against all tested cancer cell lines, including drug-sensitive HepG2, SMMC-7221, PC-3, A549, K562, HCT116, SKOV3, and MCF-7, drug-resistant HCT-8/T (resistant to paclitaxel) and HCT-8/V (resistant to vincristine) cancer cells. The growth of tumors in hybrid-treated mice has been shown to be inhibited in an in vivo HepG2 hepatocarcinoma mouse model. Moreover, these molecules markedly induce cell cycle arrest in the G2/M phase and inhibit the polymerization of tubulin [91,92]. Yan et al. also reported indole-chalcone derivatives (**43**, IC_50_: 45–782 nM; **44**, IC_50_: 23–77 nM; **45**, IC_50_: 3–679 nM) that exhibit potent antiproliferation activity in A549, HeLa, Bel-7402, MCF-7, A2780, and HCT-8 cancer cells. A further mechanistic study demonstrated that this hybrid induces low levels of cytotoxicity in normal human cells and exhibit metabolic stability in mouse liver microsomes. It also can inhibit tumor growth in xenograft models in vivo without apparent toxicity. Cellular mechanism studies elucidated that this hybrid is a novel tubulin polymerization inhibitor that binds to the colchicine site, arresting the cell cycle in the G2/M phase and inducing apoptosis along with a decrease in the mitochondrial membrane potential [93]. Cong et al. explored the anticancer potential of indole-chalcone derivatives (**46**, FC77, GI_50_: 1–53.4 nM) against a panel of MDR cancer cell lines, including HL60/DOX (doxorubicin-resistant), K562/HHT300 (homoharringtonine-resistant), CCRF-CEM/VLB100 (vinblastine-resistant), A549/T (paclitaxel-resistant), A549/DDP (cisplatin-resistant) and HCT-116/L (oxaliplatin-resistant) cells. Several of these MDR cancer cell lines show no resistance to these indole-chalcone derivatives. Further investigation revealed that this molecule can arrest cells that are involved in tubulin-binding and inhibit microtubule dynamics [94]. Taken together, these studies show that chalcone-indole hybrids can potentially serve as novel microtubule-targeting agents for the further development of potential drug candidates for the treatment of MDR cancers.

In addition to the aforementioned chalcone hybrids, other hybrids, such as chalcone–ferrocene [95,96,97], chalcone–furan/thiophene hybrids [98,99,100,101], chalcone–pyridine/pyrimidine hybrids [102,103,104,105,106], chalcone–quinoline/quinolone hybrids [107,108,109,110,111], chalcone–quinoxaline/quinazolinone hybrids [112,113,114], chalcone-quinone hybrids [115,116,117], chalcone–triazine hybrids [118,119], and chalcone–dithiocarbamate hybrids [120,121] also showed certain anticancer activity.

## 3. Representative Mechanisms of Anticancer Action of Chalcones

**Figure 10:**
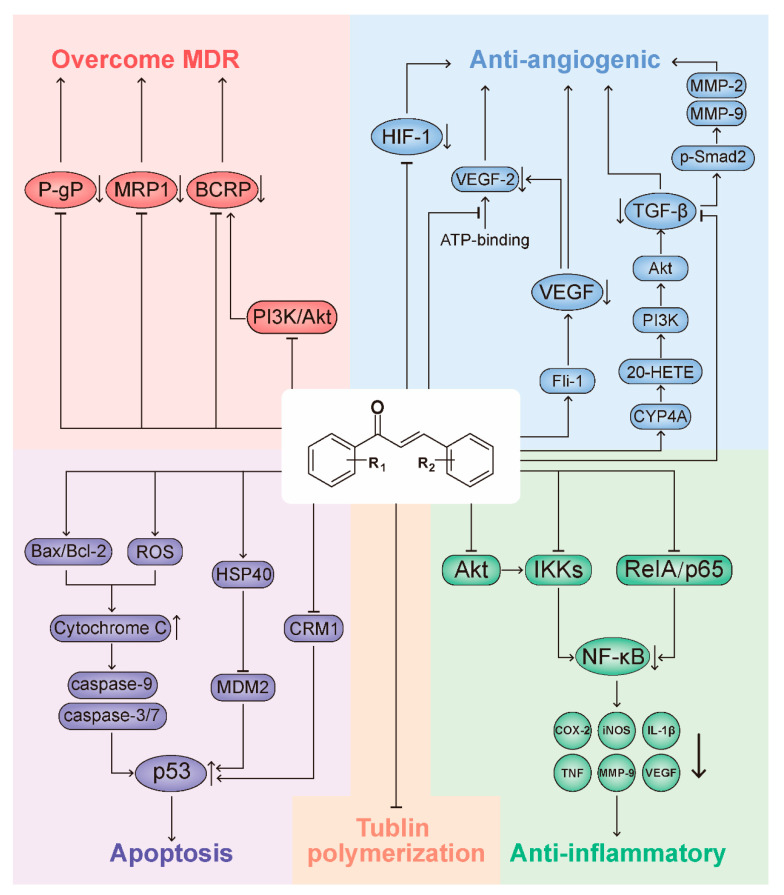
Representative Mechanisms of Anticancer Action of Chalcones. Chalcone compounds have a chemical scaffold that can be conveniently modified to alter their overall biological activity. In different screening assays, chalcones have been able to target multiple cellular molecules, such as MDM2/p53, tubulin, NF-kappa B, VEGF, VEGFR-2 kinase, HIF-1, MMP-2/9 and P-gp/MRP1/BCRP. As a result, chalcones may play an anticancer role through tumor cell apoptosis induction, microtubule polymerization, anti-inflammatory, antiangiogenesis and MDR inhabitation. This property makes chalcones very attractive as basic building blocks for the synthesis of cancer molecule-targeting agents. Abbreviations: 20-HETE, 20-Hydroxyeicosatetraenoic acid; AKT, protein kinase B; BCRP, breast cancer-resistance protein; COX-2, cyclooxygenase-2; CRM1, chromosome region maintenance 1; CYP, cytochrome P450; Fli-1, friend leukemia integration-1; HIF-1, hypoxia-inducible factor-1; HSP40, heat-shock protein 40; IKKs, IκB kinases; IL-1β, interleukin-1β; iNOS, inducible nitric oxide synthase; MDM2, the mouse double minute 2; MDR, multidrug resistance; MMP, matrix metalloproteinase; MRP1, multidrug resistance-associated protein 1; NF-κB: nuclear factor kappa-light-chain-enhancer of activated B cells; P-gp, P-glycoprotein; PI3K, phosphatidylinositol 3-kinase; p-Smad2, phosphorylation of the transcription factors Smad2; ROS, reactive oxygen species; TGF-β, transforming growth factor-β; TNF, tumor necrosis factor; VEGF, vascular endothelial factor.

Many efforts have been made to characterize the mechanism of action of chalcone compounds. Their multitarget and broad-spectrum biological activities have been reviewed in previous papers [2,6,7,8,19,122,123,124]. On the other hand, the extensive biological activity spectrum of chalcones also indicates their potential for confounding targeted treatment, posing a challenge to chalcone clinical development. Thus, understanding the mechanisms of chalcones and their direct molecular targets is particularly important for the development of clinically useful chalcone compounds in the future. This section, therefore, presents a summarizes of the representative mechanisms of the anticancer action of chalcones reported in recent years (Figure 10).

### 3.1. Chalcones Target the p53 Pathway

The tumor protein p53 modulates the cell cycle and functions as a tumor suppressor. p53 plays important roles in maintaining cellular and genomic integrity and preventing the proliferation of incipient cancer cells. p53 is degraded by various compounds, such as the mouse double minute 2 (MDM2) and Sirtuin-1. Inhibition of p53 protein degradation is a critical strategy in anticancer therapy (Table 1) [125,126].

**Figure 11:**
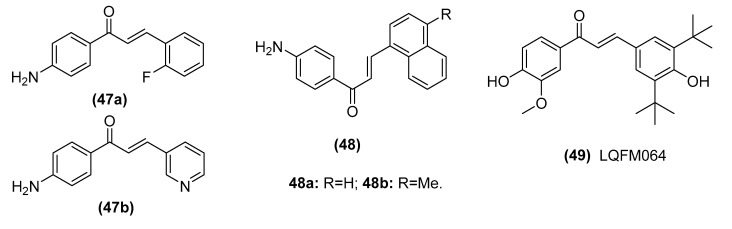
Chalcones target the p53 pathway.

MDM2 is the most notable negative regulator of p53. MDM2, an E3 ubiquitin ligase, downregulates p53 activity by constantly monoubiquitinating p53, which is an important step in mediating its degradation by nuclear and cytoplasmic proteasomes [127]. Thus, disruption of p53-MDM2 is the key event in p53 activation. Silva et al. found that trans-chalcone (TChal) (**1**) could downregulate Specificity proteins 1 (Sp1), a transcription factor involved in cell proliferation and differentiation, and upregulate p53 expression in U2OS osteosarcoma cells [128]. However, Dos et al. reported that series of chalcones with halogens on ring B and additional benzene rings (**47** IC_50_: 13.2–34.7 μM, Figure 11) showed antiproliferative activity against breast cancer cells (MCF-7 and MDA-MB-231), with upregulation of p53 and showed no effect on Sp1 protein expression [129]. Further mechanistic studies demonstrated that TChal could bind and degrade chromosome region maintenance 1 (CRM1), a nuclear export receptor involved in the active transport of tumor suppressors, and increase heat-shock protein 40 (HSP40) expression in U2OS osteosarcoma cells; the interaction of HSP40 with MDM2 blocked MDM2-mediated ubiquitination of p53, leading to the enhanced stability and activation of p53 [128,130]. Moreover, Siqueira et al. reported that TChal could reestablish the p53 pathway and prevent the overexpression of Wnt/β-catenin tumor development, inducing autophagy-related cell death and decreasing the metastatic capacity of HCC HuH7.5 cells [131]. Seba et al. reported that the chalcone derivative 4′-aminochalcone (**48**) inhibited the migration and invasion of osteosarcoma cells through the inhibition of extracellular matrix enzymatic degradation and the modulation of p53, regulating the epithelial-mesenchymal transition (EMT)-related genes [132].

A coumarlcone hybrid (**39**, S009-131) synthesized by Sashidhara et al. could induce the DNA damage response and trigger p53 activation through the phosphorylation of its key residues. Further mechanistic studies showed that the p53-MDM2 interaction was disrupted by DNA damage-induced phosphorylation. In addition, docking studies demonstrated that this hybrid could occupy the p53-binding pocket of MDM2 and increase cellular p53 levels [81,82]. Interestingly, Cabral et al. reported that a novel chalcone-like compound (**49**, LQFM064) exhibited cytotoxic activity against MCF7 cells (IC_50_: 21 μM) by inducing cell cycle arrest at the G0/G1 phase with increased p53 and p21 expression. However, the compound did not interfere directly with p53/MDM2 complexation in MCF7 cells but activated both apoptotic pathways via the modulation of proteins involved in the extrinsic and intrinsic pathways, exhibiting an increase in tumor necrosis factor receptor-1 (TNF-R1), Fas ligand (Fas-L) and Bax levels and a reduction in Bcl-2 expression [133]. Taken together, these studies suggested that the diverse chemical structures of chalcones may contribute to their differential effects mediated through the p53 pathway.

**Table 1:** Chalcones and the p53 pathway.

Lead Compounds	Mechanisms of Action	Reference
Trans-chalcone (**1**)	Enhances the expression of HSP40 and inhibits CRM1, thereby blocking MDM2-mediated ubiquitination of p53 and enhancing p53 accumulation in the nucleus.	Silva et al. [128,130]
2-fluoro-4’-aminochalcone (**47a**) and 3-pyridyl-4’-aminochalcone (**47b**)	Induces apoptosis by up-regulating p53 expression and without Sp1 expression alteration in MCF-7 cells	Dos Santos et al. [129]
Trans-chalcone (**1**)	Induces death by autophagy mediated by p53 up-regulation and Wnt/β-catenin down-regulation on human hepatocellular carcinoma HuH7.5 cell line	Siqueira et al. [131]
Chalcone derivative 4′-aminochalcone (**48**)	Suppresses migration and invasion of osteosarcoma cells mediated by p53 regulating EMT-related genes	Seba et al. [132]
Coumarlcone hybrid (**39**, S009-131)	Instigates DNA damage, disrupts p53-MDM2 interaction and stabilizes p53 through post-translational modifications in both vitro and vivo of HeLa cells	Sashidhara et al. [81,82]
(*E*)-3-(3, 5-di-ter-butyl-4-hydroxyphenyl)-1-(4-hydroxy-3-methoxyphenyl) prop-2-en-1-one (**49**, LQFM064)	Induces cell cycle arrest at the G0/G1 phase with up-regulation of p53 and p21	Cabral et al. [133]

### 3.2. Chalcones Target Tubulin Polymerization

Tubulin is a dimeric protein consisting of two similar, nonidentical subunits: α and β. The discovery of chalcones as antimitotic agents was first reported nearly 30 years ago [134]. Based on the understanding of the colchicine-tubulin structure–activity relationship, chalcone derivatives are modeled as colchicine analogs, which can bind to tubulin and prevent its polymerization, leading to abrupt interruption of mitotic spindle assembly, interference with the function of the cytoskeleton, and interrupted mitosis. The structure–activity relationships of numerous chalcone derivatives have since been studied with colchicine and vinblastine as controls (Table 2).

**Figure 12:**
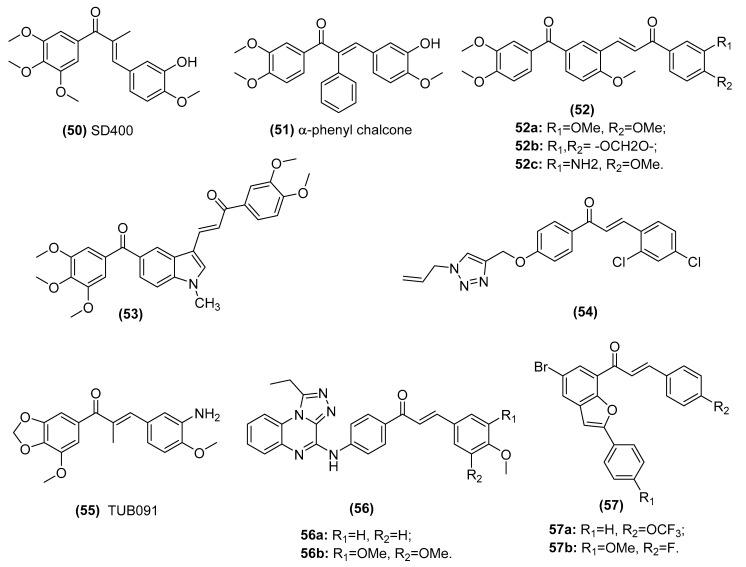
Chalcones target tubulin polymerization.

Numerous chalcones have demonstrated a capacity for binding β-tubulin similar to that of combretastatin 4A, destabilizing microtubule polymers and thus serving as combretastatin 4A analogs. Ducki et al. synthesized and developed CA-4 type chalcones SD400 (**50**) and α-phenyl chalcone (**51**) (Figure 12), which displayed potent inhibition of tubulin polymerization with arrest in the G2/M phase and showed promising antivascular activity [135,136]. Kamal et al. reported that a series of phenstatin/isocombretastatin–chalcones (**52**, GI_50_: 0.11–19.0 μM) displayed potent antiproliferative activity in a panel of sixty human cancer cell lines [137]. A competitive binding assay suggested that these compounds were bound to the colchicine-binding site of tubulin, inducing potent inhibitory effects on tubulin assembly and leading to arrest in the G2/M phase and apoptotic cell death. Moreover, Kode et al. found that new phenstatin-based indole-linked chalcone compounds (**53**, GI_50_ < 0.1 µM) showed anticancer activity in SCC-29B human oral cancer cells, spheroids and AW13516 oral cancer xenograft model mice [138]. Further mechanistic studies revealed that these compounds destabilized tubulin, leading to loss of cell integrity and affecting glucose metabolism. In addition, surface plasmon resonance confirmed direct molecular interactions between tubulin and these compounds. A novel chalcone-1,2,3-triazole derivative (**54**) synthesized by Yan et al. showed significant antitumor activity against liver cancer HepG2 cells in vitro with an IC_50_ value of 0.9 μM and in vivo with low toxicity in mice. Moreover, they found that this derivative might be a potent tubulin polymerization inhibitor [139]. Canela et al. reported a new chalcone, called TUB091 (**55**), that binds to the colchicine site of tubulin, as shown by X-ray crystallography [140]. TUB091 inhibited cancer and endothelial cell growth and induced G2/M phase arrest and apoptosis at 1-10 nM. Furthermore, TUB091 showed vasculature disruption both in vitro and in melanoma and breast cancer xenograft models. Alswah et al. reported that triazoloquinoxaline-chalcone derivatives (**56**, IC_50:_ 1.65–34.28 µM) displayed significant antiproliferative effects against MCF-7, HCT-116 and HEPG-2 cells [141]. A molecular docking study demonstrated that the binding modes of these chalcones were targeted to epidermal growth factor receptor tyrosine kinase (EGFR-TK) and tubulin. Moreover, Mphahlele and his coworkers found that 2-arylbenzo[*c*]furan-chalcone hybrids (**58**) showed the potential to exhibit inhibitory effects against tubulin polymerization and EGFR-TK phosphorylation [98]. In summary, chalcones can be considered attractive tubulin polymerization inhibitor candidates for developing anticancer therapeutics.

### 3.3. Chalcones and the NF-κB Pathway

The nuclear factor kappa B (NF-κB) is a crucial transcription factor that plays an important role in inflammation, innate immunity and carcinogenesis. NF-κB signaling was shown to contribute to cancer progression through the upregulation of tumor-promoting cytokines and survival genes (e.g., Bcl-2), inhibition of apoptosis, and promotion of angiogenic factors, and it manifests a migratory and invasive phenotype [142]. Thus, NF-κB inhibitors mediated effects that could lead to an antitumor response or to moresensitive antitumor drug action. NF-κB activity is regulated primarily by IκB kinases (IKKs), which are recognized as the central mediators of immune responses and inflammation [143]. Interference of NF-κB function through IKK inhibition is expected to suppress NF-κB protein translocation to the nucleus and is considered a promising strategy for disease treatment, especially against inflammation and inflammation-related cancer [144,145,146].

**Figure 13:**
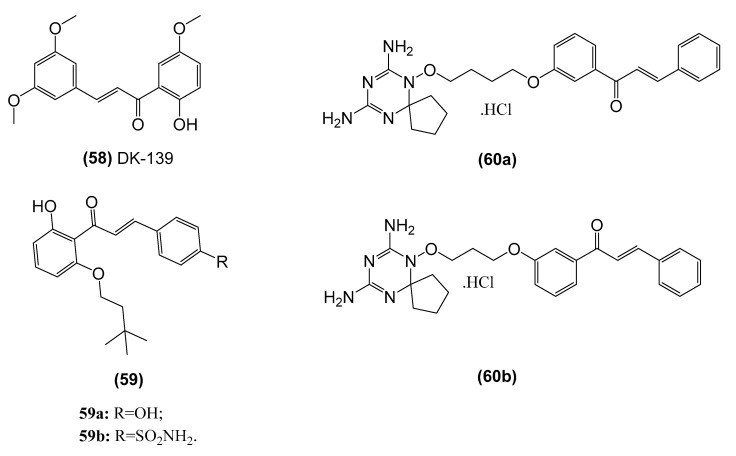
Chalcones and the NF-κB pathway.

Chalcones demonstrate NF-κB inhibitory activity by inducing the covalent modification of IKK proteins via an α,β-unsaturated ketone with Michael-type activity (Table 3). Indeed, Pandey et al. reported that butein (3,4,2’,4’-tetrahydroxy chalcone) (**7**) suppressed NF-κB activity and NF-κB-regulated gene expression by conjugating the cysteine 179 residue of IKK and then blocking the phosphorylation and degradation of IκBα [147]. Similarly, 2-hydroxy-3’,5,5’-trimethoxychalcone (DK-139) (**58**, Figure 13) inhibited the Toll-like receptor 4-mediated inflammatory response through suppression of the Akt//IKK/NF-κB signaling pathway in BV2 microglial cells [148]. In addition, isoliquiritigenin [149,150,151], flavokawain A and B [152], licochalcone A [153], cardamonin [154,155,156] and xanthohumol [157] all showed anti-inflammatory and anticancer activities by preventing the degradation of IκBα and in turn blocking NF-κB activation. Moreover, Venkateswararao et al. evaluated the inhibitory activity of a series of chalcone derivatives (**59**, IC_50_: 1.17–3.43 µM) against NF-κB and their in vitro antiproliferative activity against various human cancer cell lines, and they found a good correlation between NF-κB inhibition through these derivatives and antiproliferative activity [158]. Gan et al. also designed two novel dihydrotriazine-chalcone compounds (**60**) previously found to exert antiproliferative effects through dual targeting of dihydrofolate reductase (DHFR) and TrxR, which could inhibit the in vitro migration of MDA-MB-231 breast carcinoma cells through dose-dependent downregulation of matrix metalloproteinase-9 (MMP-9) expression and secretion [159]. Moreover, these chalcone-based compounds inhibited inflammatory mediator expression, including inducible nitric oxide synthase (iNOS) and cyclooxygenase-2 (COX-2) and tumor necrosis factor alpha (TNF-α), through suppression of the NF-κB signaling pathway. In conclusion, chalcones can be developed as novel scaffolds that target both invasion and inflammation to develop chemopreventive and/or anticancer therapies.

### 3.4. Chalcones as Inhibitors of Angiogenesis

Angiogenesis is a promising target for cancer treatment due to its important role in cancer progression and metastasis. Chalcones were recently documented to modulate several steps in angiogenesis, such as vascular endothelial factor (VEGF), basic fibroblast growth factor (bFGF), transforming growth factor-β (TGF-β) signaling pathway or hypoxia-inducible factor-1 (HIF-1) [160,161,162], MMP activity or endothelial cell proliferation and migration (Table 4).

**Figure 14:**
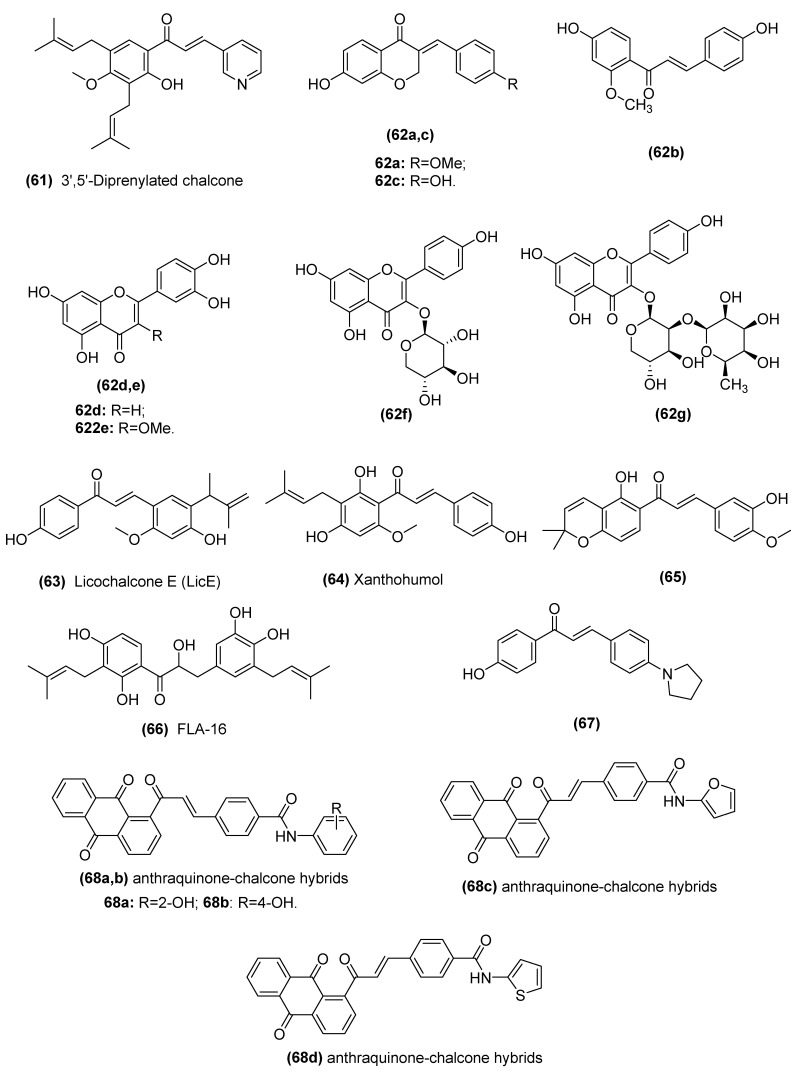
List of chalcones as inhibitors of angiogenesis.

Ma et al. found that a 3′,5′-diprenylated chalcone (**61**, Figure 14) likely showed friend leukemia integration-1 (Fli-1) agonism and regulation of the expression of VEGF-1, TGF-β2, intercellular cell adhesion molecule-1 (ICAM-1), p53, and MMP-1 genes, which are associated with tumor apoptosis, migration, and invasion in prostate cancer cells [163]. Iheagwam et al. performed a molecular docking analysis, also showing that flavonoids (**62**) isolated from young *Caesalpinia bonduc* twigs and leaves displayed strong interactions with TK, VEGF, and MMP [164]. Wang et al. screened nearly 36,043 compounds and found that isoliquiritigenin (**6**) exhibited the most potent antiangiogenic activities in zebrafish with the concentration for 50% of maximal effect (EC_50_) values of 5.9 μM [28]. Mechanistically, isoliquiritigenin inhibited arachidonic acid (AA) metabolic enzymes, including COX-2, microsomal prostaglandin E synthase-1 (mPGES-1) and cytochrome P450 (CYP) 4A11, in glioma and resulted in the inhibition of the angiogenic Akt-FGF-2/TGF-β/VEGF signaling pathway through ceRNA effects on miR-194-5p and lncRNA NEAT1. Moreover, Wang et al. also found that isoliquiritigenin could inhibit breast cancer growth and neoangiogenesis accompanied by VEGF/VEGFR-2 signaling suppression, an elevated apoptosis rate and few toxicity effects both in vitro and in vivo [165]. Molecular docking simulation indicated that isoliquiritigenin could stably form hydrogen bonds and aromatic interactions within the ATP-binding region of VEGFR-2. Kwon et al. demonstrated that the oral administration of licochalcone E (LicE) (**63**) significantly inhibited solid tumor growth and lung metastasis of mammary cancer cells, which was associated with suppression of tumor angiogenesis and lymphangiogenesis, as well as a decrease in inflammatory status in the tumor and lung (the target organ) tissues [166]. In addition, Saito et al. found that xanthohumol (**64**), a prenylated chalcone in hops (*Humulus lupulus* L.), blocked angiogenesis in pancreatic cancer by reducing both NF-κB activity and the subsequent production of angiogenic factors VEGF and IL-8 in vitro and in vivo [167]. 

In addition to the inhibitory effect of chalcones on VEGF, chalcones also demonstrated potential inhibitory activity against other angiogenic factors. Wang et al. synthesized a novel series of chalcone derivatives (**65**), and a tested compound exhibited significant inhibitory effects on HIF-1. In addition, the tested compound inhibited tumor invasion and angiogenesis in vitro and in vivo with good tolerance and presented a good therapeutic window [168]. Wang and colleagues reported that FLA-16 (**66**), a novel chalcone-type flavonoid isolated from *Glycyrrhiza glabra* roots, inhibited CYP4A, prolonged the survival and normalized the vasculature of glioma by decreasing the production of tumor-associated macrophages (TAMs) and endothelial progenitor cell (EPC)-derived VEGF and TGF-β in vitro and in vivo [169]. Jeong et al. also reported that an improved analog of chalcone (**67**) could suppress the TGF-β1-induced EMT of human A549 lung cancer cells [170]. Stanojkovic et al. found a series of highly selective anthraquinone–chalcone hybrids (**68**, IC_50_: 3.87–5.99 μM) showing anti-invasive, antimetastatic, and antiangiogenic properties by decreasing the expression levels of MMP2, MMP9, and VEGF against K562 cells [171]. Moreover, a number of chalcone analogs of combretastatin 4A demonstrated significant effects in tumor blood vessels (for details, see Section 3.2). In summary, the use of chalcones is a potential therapeutic strategy to reduce inflammation and tumor angiogenesis.

### 3.5. Chalcones as Inhibitors of MDR Channels

Numerous studies have focused on the ability of chalcone to mediate resistance to conventional chemotherapeutic drugs by modulating multidrug efflux transporters, which are essential components of intracellular drug accumulation [172,173]. Most of the investigated chalcones showed the ability to inhibit three well-characterized members of the ATP-binding cassette (ABC) transporter family: P-glycoprotein (P-gp, ABCB1), multidrug resistance-associated protein 1 (MRP1, also known as ABCC1), and breast cancer-resistance protein (BCRP, ABCG2), main contributors to the MDR in cancer cells. Therefore, chalcones have received considerable attention as chemosensitizers for clinical drug resistance and for improving the pharmacokinetics of poorly absorbed chemotherapeutic cancer drugs (Table 5).

**Figure 15:**
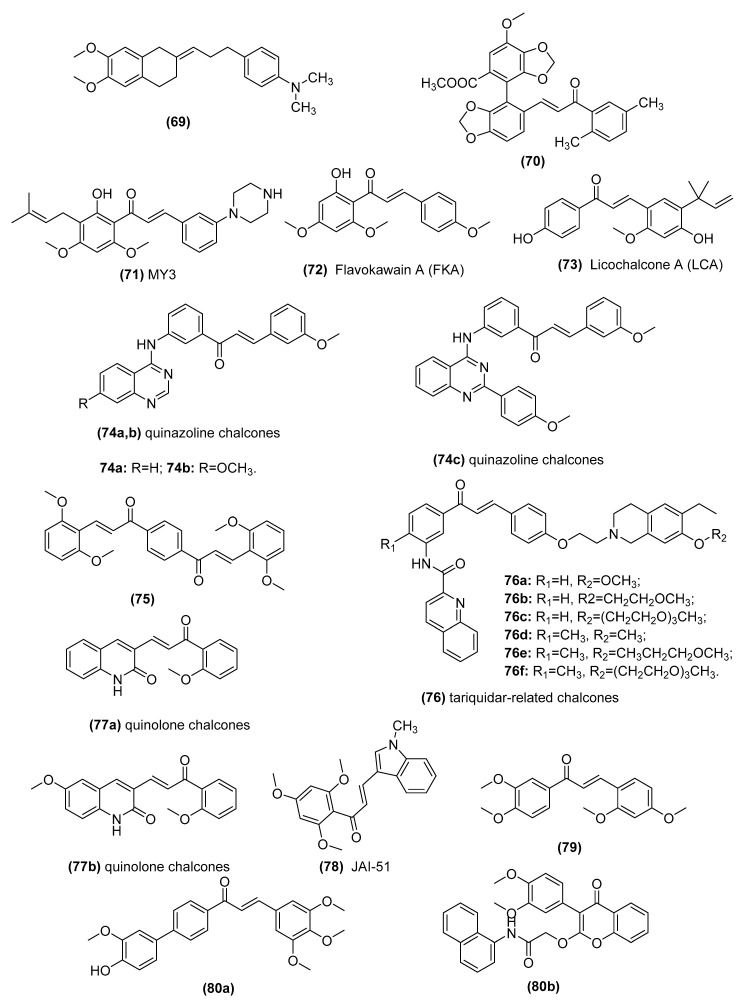
List of chalcones as inhibitors of MDR Channels.

In the late 1990s, Bois et al. explored the structural requirements of chalcones for P-gp regulation and found that 2′,4′,6′-trihydroxy-4-iodochalcone and 4-alkoxy-2′,4′,6′-trihydroxychalcones exhibited high-affinity binding for P-gp [174,175]. Recently, using computer-aided drug design (CADD) methods, including 2D- and 3D-quantitative structure–activity relationships (QSAR), molecular modeling, and docking analyses, Parveen et al. [176] and Ngo et al. [177], reported a series of novel synthesized chalcones (**69**, IC_50_: 42 nM; Figure 15) showing high levels of biological activity and indicating the importance of specific groups for P-gp inhibitory effects. Other authors explored the ability of chalcone derivatives to regulate the effects of P-gp on MDR. For example, Gu and colleagues synthesized a series of bifendate–chalcone hybrids (**70**) and found that the best compound with minimal intrinsic cytotoxicity (IC_50_ > 200 μM) could increase the accumulation of rhodamine-123 in K562/A02 human leukemia cells more potently than bifendate or verapamil (VRP) by inhibiting P-gp efflux function [178]. Moreover, Yin et al. also reported that a novel chalcone derivative, MY3 (**71**, IC_50_: 1.02 μM), not only induced little intrinsic cytotoxicity but also reversed DOX resistance in MCF-7/DOX cells via suppression of P-gp [179]. More importantly, MY3 markedly enhanced the efficacy of DOX treatment in MCF-7 tumor xenografts, without changed model body weight. Li et al. reported that flavokawain A (FKA) (**72**, IC_50_: 21 µM) inhibited P-gp protein expression by blocking the PI3K/Akt pathway in paclitaxel (PTX)-resistant A549 (A549/T) cells, indicating that FKA combined with PTX reversed PTX resistance [180]. Furthermore, they used national medical product administration (NMPA)-approved aescinate (Aes) to prepare twin-like nanoparticles (NPs) such as PTX-A NPs and FKA-A NPs, which reversed PTX resistance both in vitro and in vivo by inhibiting P-gp expression in A549/T cells [181].

Moreover, the potential modulatory effect of bioactive compounds of chalcones on ABCG2-mediated multidrug resistance has been investigated for years. Wu et al. reported that licochalcone A (LCA) (**73**), a natural chalcone isolated from the root of *Glycyrrhiza inflata*, inhibited the drug transport function of ABCG2 and reversed ABCG2-mediated multidrug resistance in human multidrug-resistant cancer cell lines in a concentration-dependent manner [182]. An in silico docking analysis used to discern the role of LCA in the inward-open conformation of human ABCG2 revealed LCA binding ABCG2 in the transmembrane substrate-binding pocket. Kraege et al. synthesized a series of novel heterodimeric modulators based on the combination of chalcones with quinazolines (**74**), and the most potent ABCG2 inhibitor in this series (IC_50_: 0.19 μM) was selective, nontoxic (GI_50_: 93 μM) and able to reverse MDR [114]. Winter and colleagues synthesized symmetric bis-chalcones with different substituted groups and screened them to determine their ability to inhibit mitoxantrone efflux from ABCG2-transfected HEK293 cells. The best derivative (**75**) was selective for ABCG2 over P-glycoprotein and MRP1 and stimulated ABCG2 basal ATPase activity [183]. Peña-Solórzano et al. found that tariquidar-related chalcones (**76**) exhibited selectivity for ABCG2 to a greater extent than the transporters ABCB1 and ABCC1 [109]. Notably, novel quinolone chalcones (**77**) synthesized by Lindamulage et al. could overcome multidrug resistance by impeding MRP1 function while maintaining strong inhibition of microtubule activity [111]. This compound exhibited strong anticancer activity, alone or in combination with paclitaxel, without causing any notable side effects in mice engrafted with MDA-MB-231 triple-negative breast cancer cells.

Interestingly, some studies demonstrated the effect of a dual ABCG2/ABCB1 inhibitor. For example, Boumendjel et al. reported a novel chalcone derivative, JAI-51 (**78**), acting not only as a microtubule-depolymerizing agent but also as an inhibitor of P-gp and BCRP in vitro and in vivo in glioblastoma models [184]. Han et al. [185] and Cai et al. [186] found that nonbasic chalcone (**79**) served as a dual ABCG2/ABCB1 inhibitor by inhibiting ABCG2 and ABCB1 ATPase activities, not by altering the expression or localization of the ABCG2 or ABCB1 transporters. Silbermann et al. described two novel chalcone and flavone derivatives (**80**) [99]. One of these inhibitors (**80a**, IC_50_: 3.37 μM) was a highly potent reverser of ABCG2-mediated MDR and a parallel dual ABCC1/ABCG2 inhibitor. Another was a highly potent dual ABCB1/ABCG2 MDR reverser. In summary, chalcones might be promising lead compounds to develop as chemosensitizers for clinical drug resistance, as well as for improving the pharmacokinetics of poorly absorbed chemotherapeutic cancer drugs.

**Table 5:** Chalcones as inhibitors of MDR channels.

Lead Compounds	Mechanisms of Action	Reference
2-[3-(4-Dimethylaminophenyl)-prop-2-en-yliden]-5,6- dimethoxyindan-1-one (**69**)	Inhibitors of human P-glycoprotein	Parveen et al. [176] and Ngo et al. [177]
Bifendate chalcone hybrids (**70**)	P-gp inhibitors in K562/A02 cells whichoverexpress P-gp (induced by adriamycin)	Gu et al. [178]
MY3 (**71**)	Inhibits expression of P-gp and enhances the efficacy of DOX against the tumor xenografts bearing MCF-7/DOX cells	Yin et al. [179]
Flavokawain A (FKA) (**72**)	Inhibits P-gp protein expression by blocking the PI3K/Akt pathway in PTX-resistant lung cancer A549 cells	Li et al. [180,181]
Licochalcone A (LCA) (**73**)	Binds ABCG2 in the transmembrane substrate-binding pocket and reverses ABCG2-mediated multidrug resistance in human multidrug-resistant cancer cell lines	Wu et al. [182]
Quinazoline chalcones (**74**)	Modulators of breast cancer resistance protein (BCRP/ABCG2) in P-gp overexpressing MDCK II cells	Kraege et al. [114]
Symmetric bis-chalcones (**75**)	Inhibits mitoxantrone efflux from ABCG2-transfected HEK293 cells by stimulating ABCG2 basal ATPase activity	Winter et al. [183]
Tariquidar-related chalcones (**76**)	ABCG2 Modulators in ABCG2-overexpressing MCF-7/Topo cells	Peña-Solórzano et al. [109]
Quinolone chalcones (**77**)	Targets colchicine-binding pocket and kills multidrug-resistant cancer cells by inhibiting tubulin activity and MRP1 function	Lindamulage et al. [111]
A novel chalcone derivative, JAI-51 (**78**)	A microtubule-depolymerizing agent and an inhibitor of P-gp and BCRP in vitro and in vivo of glioblastoma models	Boumendjel et al. [184]
Nonbasic chalcone (**79**)	A dual ABCG2/ABCB1 inhibitor in S1-M1-80 (mitoxantrone (MX)-selected ABCG2-overexpressing of human colorectal cancer cell line S1), NCI-H460/MX20 (MX-selected ABCG2-overexpressing of human lung cancer cell line NCI-H460)	Han et al. [185] and Cai et al. [186]
Novel chalcone and flavone derivatives (**80**)	Selective and dual inhibitors of the transport proteins ABCB1 and ABCG2 in ABCG2-overexpressing MDCK II BCRP cells	Silbermann et al. [99]

### 3.6. Other Molecular Cancer Targets Modulated by Chalcones and Target Identification

Chalcones have been shown to exhibit anticancer activity through their inhibitory potential against various targets, such as 5α-reductase [187], aromatase [188], histone deacetylase inhibitors (HDAC) [189,190], proteasome [191,192], JAK/STAT signaling pathways [193], cell division cycle 25 (CD25) [194], cathepsin-K [72,195], topoisomerase-II [196,197], Wnt [198,199], ROS/MAPK [200], p38 [201,202] and mTOR [203,204]. However, these studies are not covered in this review because not enough evidence has been provided to indicate that these proteins or pathways are direct targets of chalcones. Therefore, the identification and confirmation of the molecular target(s) of chalcones are important steps in pharmaceutical research [205]. Researchers have invested tremendous effort to explore direct-binding targets of chalcones using a variety of strategies. We discuss the most commonly used experimental methods for target recognition, such as computer strategies.

Computer strategies, such as QSARs assessments, molecular docking, and virtual screening, have been widely used in research aimed to find the targets of natural and synthetic chalcones. Marquina et al. designed and synthesized eleven 4′-alkoxy chalcones that could induce the mitochondrial apoptotic pathway by regulating Bax and Bcl-2 transcripts and by increasing caspase 3/7 activation. This QSAR model study revealed that the double bond of the α,β-unsaturated carbonyl and the planar structure geometry was important to the biological activity of synthetized chalcones [206]. Song et al. discovered human carboxylesterase 2 (hCES2A) inhibitors obtained from *Glycyrrhiza inflata* through a combination of docking-based virtual screening and fluorescence-based inhibition assays [207]. hCES2A is a key target to ameliorate the intestinal toxicity triggered by irinotecan, which causes severe diarrhea in 50–80% of patients receiving this anticancer agent. Following the screening of 73 herbal products, Song et al. found that licochalcone C, chalcones and isolicoflavonol in licorice were the key compounds critical for hCES2A inhibition, which will be very helpful in developing new herbal remedies or drugs for ameliorating hCES2A-associated drug toxicity. Molecular docking with corresponding chalcones has also been applied to predict the binding mode and explain the phenotypic activity of EGFR [103,208], aurora kinase [209,210], anaplastic xanthine oxidase (XO), the colchicine binding site of the tubulin [211], the estrogen receptor [212,213] and acetylcholinesterase (AChE) [214]. The advantage of using a computational strategy is the convenience of predicting the binding target(s) of chalcones before biological validation. However, the binding site and binding specificity need to be validated.

## 4. Summary and Perspectives

Chalcones play central roles in the flavonoid synthesis pathway and are ubiquitous in many natural products. Chalcones exhibit a broad spectrum of biological activity, probably due to their small structure and Michael-acceptor features, making them tolerant of different biological molecules, allowing their ready or reactive binding with certain molecules. In addition, chalcone compounds have a chemical scaffold that can be conveniently modified to alter their overall biological activity. In different screening assays, chalcones have been able to target multiple cellular molecules, such as MDM2/p53, tubulin, NF-κB, ABCG2/P-gp/BCRP, VEGF, VEGFR-2 kinase, and MMP-2/9.

Despite medicinal applications of chalcones, their wide bioactivity spectrum indicates a potentially promiscuous targeting profile, which presents a challenge for clinical development. Recently, Xing and colleagues found a hepatotoxic risk for flavokawain A (58) and B (59) (Figure 4), two chalcone derivatives isolated from kava used for their anxiolytic and sedative effects and that show hepatotoxic synergism with acetaminophen [215]. Using in silico tools, Tugcu et al. also illustrated the hepatotoxicity potential of kava [216]. According to the experimental results and predictions, kava constituents play substantial roles in hepatotoxicity by mechanisms involving glutathione depletion, CYP inhibition, reactive metabolite formation, mitochondrial toxicity and/or cyclooxygenase activity. Moreover, Qian et al. reported that a licorice monomer compound (2′-HC) at a concentration of ≥20 µM resulted in excess ROS production and inadequate Cu/Zn Superoxide Dismutase (SOD1) expression in hepatocytes [217]. Therefore, the design and synthesis of new analogs are particularly important for the future development of clinically useful chalcone derivatives. Computer strategies and CADD approaches represent time-, labor-, and cost-effective strategies for identifying lead compounds in the early stages of drug discovery.

Notably, metochalcone has been approved as a choleretic drug [2], and sofalcone is an anti-ulcer agent that increases the amount of mucosal prostaglandin, conferring a gastroprotective effect against *Helicobacter pylori* [8]. These drugs represent a promising strategy for developing chalcones as novel anticancer agents. Recently, Jeong et al. [218] and Oh et al. [219] have shown that synthetic chalcones can have a heat-shock protein 90 (HSP90)-induced inhibitory effect, which opens new perspectives into cancer treatment through the destabilization proteins by which cancer cells survive and multiply (tumorigenesis) [220]. Several phase II clinical trials of new anticancer molecules that have two hydroxyl groups at positions 1 and 3 have revealed the inhibition of interactions between HSP90 and patient proteins through the binding of these molecules to the ATP site in HSP90 [221,222]. Perhaps in the future, phase III clinical trials will be conducted to support the anticancer potential of chalcones and their derivatives. Interestingly, Song et al. have shown that the combining licochalcone C with irinotecan can ameliorate the intestinal toxicity induced by irinotecan by suppressing hCES2A [207]. Therefore, the combination of chalcones and other therapies is expected to be an effective way to improve anticancer therapeutic efficacy.

Moreover, for more than half a century, global marine sources have proven to be a rich source of a vast array of new medicinally valuable compounds [223,224]. However, there is a lack of research on chalcones in marine natural products. De Luca et al. analyzed the microalgal transcriptomes available in the Marine Microbial Eukaryotic Transcriptome Sequencing Project (MMETSP) database for an in silico search of chalcone synthase and 4-Coumarate: CoA ligase, an upstream enzyme in the synthesis of chalcones [225]. They first report the occurrence of these enzymes in specific microalgal taxa. This study gives new insights into possible biotechnological applications for the production of bioactive compounds, such as chalcones.

In general, chalcones are easy to chemically modify and synthesize to generate compounds with a wide variety of structural diversities. This property makes chalcones very attractive as basic building blocks for the synthesis of molecule-targeting agents. In the coming era of molecularly targeted therapy and personalized medicine, both naturally occurring and synthetic chalcones may be very useful tools for studying basic mechanisms of cancer treatment and prevention and for developing novel agents for targeted cancer therapies.

## Figures and Tables

**Table 2:** Chalcones affect tubulin polymerization.

Lead Compounds	Mechanisms of Action	Reference
CA-4 type chalcones SD400 (**50**) and α-phenyl chalcone (**51**)	Populate the colchicine-binding site of beta-tubulin and inhibit tubulin assembly in the K562 human chronic myelogenous leukemia cell line	Ducki et al. [135,136]
A series of phenstatin/isocombretastatin–chalcones (**52**)	Inhibit tubulin assembly, arrest in the G2/M phase and induce apoptosis in a panel of sixty human cancer cell lines	Kamal et al. [137]
Phenstatin based indole linked chalcone compounds (**53**)	Destabilizes tubulin, leading to loss of cell integrity and affecting glucose metabolism in SCC-29B human oral cancer cells, spheroids and AW13516 oral cancer xenograft model mice	Kode et al. [138]
Chalcone-1,2,3-triazole derivative (**54**)	Inhibits tubulin polymerization in liver cancer HepG2 cells	Yan et al. [139]
(*E*)-3-(3-amino-4-methoxyphenyl)-1-(5’-methoxy-3’,4’-methylendioxyphenyl)- 2-methylprop-2-en-1-one (**55**, TUB091)	Destabilizes microtubule, targets vascular and shows antitumor and antimetastatic activities in melanoma and breast cancer xenograft models	Canela et al. [140]
Triazoloquinoxaline-chalcone derivatives (**56**)	Displays significant antiproliferative effects against MCF-7, HCT-116 and HEPG-2 cells	Alswah et al. [141]
2-arylbenzo[*c*]furan-chalcone hybrids (**57**)	Inhibits tubulin polymerization and EGFR-TK phosphorylation in the human breast cancer (MCF-7) cell line	Mphahlele et al. [98]

**Table 3:** Chalcones and the NF-κB Pathway.

Lead Compounds	Mechanisms of Action	Reference
Butein (**7**)	Blocks the phosphorylation and degradation of IκBα and suppresses NF-κB activity in KBM-5 (human myeloid) cells	Pandey et al. [147]
2-hydroxy-3’,5,5’-trimethoxychalcone (**58**, DK-139)	Inhibits the Akt//IKK/NF-κB signaling pathway in BV2 microglial cells	Lee et al. [148]
(*E*)-1-(2-hydroxy-6- (isopentyloxy)phenyl)-3-(4-hydroxyphenyl)prop-2-en-1-one (**59**)	Inhibits NF-κB activity in vitro and shows antiproliferative activity against various human cancer cell lines, namely ACHN (renal), NCI-H23(lung), MDA-MB-231 (breast), HCT-15 (colon), NUGC-3 (stomach) and PC-3 (prostate).	Venkateswararao et al. [158]
Dihydrotriazine-chalcone compounds (**60**)	Inhibits IKKα/β phosphorylation, leading to a reduction in phosphorylation of the p65 subunit and eventually suppression of NF-κB-dependent transcriptional activation of MMP-9 expression. in MDA-MB-231 breast carcinoma cells	Gan et al. [159]

**Table 4:** Chalcones affect the tumor vasculature.

Lead Compounds	Mechanisms of Action	Reference
3′,5′-diprenylated chalcone (**61**)	An Fli-1 agonist for regulating the expression of Fli-1 target genes including VEGF-1, TGF-β2 and MMP-1 genes of prostate cancer cells	Ma et al. [163]
Flavonoids (**62**) isolated from young *Caesalpinia bonduc* twigs and leaves	A molecular docking analysis shows the interaction between them and cancer target proteins (TK, VEGF, and MMP)	Iheagwam et al. [164]
Isoliquiritigenin (**6**, ISL)	Inhibits the angiogenic Akt- FGF-2/TGF-β/VEGF signaling in C6 glioma cell line and the rat C6 glioma model.	Wang et al. [28]
Isoliquiritigenin (**6**, ISL)	Inhibits VEGF expression via promoting HIF-1α proteasome degradation pathway and blocks VEGFR-2 activation and the transduction of its downstream signaling in human umbilical vein endothelial cells (HUVECs), MCF-7 cells and MDA-MB-231 cells and in vivo tumor xenograft of MDA-MB-231 cells	Wang et al. [165]
Licochalcone E (**63**, LicE)	Decreases expression of VEGF-A and C, VEGF receptor 2, and HIF-1α in the mouse model of breast cancer	Kwon et al. [166]
Xanthohumol (**64**)	Inhibits angiogenesis by suppressing NF-κB activation and the subsequent production of angiogenic factors VEGF and IL-8 in vitro and in vivo of pancreatic cancer (BxPC-3)	Saito et al. [167]
Chalcone-based compounds with 2,2-dimethylbenzopyran (**65**)	Inhibits HIF-1 by downregulating the expression of HIF-1α in Hep3B and HUVEC cells and Hep3B xenograft models	Wang et al. [168]
2, 3’, 4, 4’-tetrahydroxy-3, 5’-diprenylchalcone (**66**, FLA-16)	Inhibits CYP4A and prolongs survival and normalizes vasculature in C6 and U87 gliomas tumor models through decreasing production of TAMs and EPCs-derived VEGF and TGF-b.	Wang et al. [169]
Analog of chalcone (**67**)	Suppresses TGF β1, induces EMT markers, MMP 2 and MMP 9, and inhibits migration and invasion of A549 cells	Jeong et al. [170]
Anthraquinone-chalcone hybrids (**68**)	Decreases in the expression levels of MMP2, MMP9, and VEGF in K562 cells	Stanojkovic et al. [171]

## Data Availability

Research data are not shared.

## Abbreviations

AA, arachidonic acid; ABC, ATP-binding cassette; AChE, acetylcholinesterase; AKT, protein kinase B; ATM, ataxia telangiectasia mutated; BCRP, breast cancer-resistance protein; bFGF, basic fibroblast growth factor; CADD, computer-aided drug design; Cat, cathepsin; CC_50_, the median cytotoxic concentration; CD25, cell division cycle 25; cdc25C: cell division cycle 25C; COX-2, cyclooxygenase-2; CRM1, chromosome region maintenance 1; CYP, cytochrome P450;DHFR, dihydrofolate reductase; DOX, doxorubicin; DR, death receptors; EC_50_, the concentration for 50% of maximal effect; EGFR, epidermal growth factor receptor; EGFR-TK, epidermal growth factor receptor tyrosine kinase; EMT, epithelial-mesenchymal transition; EPC, endothelial progenitor cell; ERKs, extracellular signal-regulated kinases; Erα, estrogen receptor alpha; Fas-L, Fas ligand; FKA, flavokawain A; Fli-1, friend leukemia integration-1; GI_50_, the concentration that causes 50% growth inhibition; GSH, glutathione; HCC, hepatocellular carcinoma; hCES2A, human carboxylesterase 2; HDAC, histone deacetylase inhibitors; HDACs, histone deacetylases; HIF-1, hypoxia-inducible factor-1; HPyCT4BrPh, (E)-3-(4-bromophenyl)-1-(pyridin-2-yl)prop-2-en-1-one thiosemicarbazone; HSP, heat-shock protein; IBC, Isobavachalcone; IC_50_, half-maximal inhibitory concentration; ICAM-1, intercellular cell adhesion molecule-1; IKKs, IκB kinases; IL-6, interleukin-6; iNOS, inducible nitric oxide synthase; ISL, Isoliquiritigenin; LCA, licochalcone A; LicE, licochalcone E; MDM2, the mouse double minute 2; MDR, multidrug resistance; MMP, matrix metalloproteinase; mPGES-1, microsomal prostaglandin E synthase-1; MRP1, multidrug resistance-associated protein 1; NF-κB: nuclear factor kappa-light-chain-enhancer of activated B cells; NMPA, national medical product administration; Nrf2, NF-E2-related factor 2; NSCLC, non-small cell lung cancer; P-gp, P-glycoprotein; PTX, paclitaxel; PUMA, p53 upregulated modulator of apoptosis; QSAR, Quantitative structure–activity relationships; ROS, reactive oxygen species; RSK2, ribosomal S6 kinase 2; SAR, structure–activity relationship; SOD1, Cu/Zn Superoxide Dismutase; Sp1, Specificity proteins 1; Src, Proto-oncogene tyrosine-protein kinase; TAMs, tumor-associated macrophages; TGF-β, transforming growth factor-β; TNBC, triple negative breast cancer;TNFR-1, tumor necrosis factor receptor-1; TrxR1, thioredoxin reductase 1; VEGF, vascular endothelial factor; XO, xanthine oxidase; TChal, trans-chalcone.

## References

[1] Hussain S., Singh A., Nazir S.U., Tulsyan S., Khan A., Kumar R., Bashir N., Tanwar P., Mehrotra R. (2019). Cancer drug resistance: A fleet to conquer. J. Cell. Biochem..

[2] Jandial D.D., Blair C.A., Zhang S., Krill L.S., Zhang Y.-B., Zi X. (2014). Molecular targeted approaches to cancer therapy and prevention using chalcones. Curr. Cancer Drug Targets.

[3] Aiello P., Sharghi M., Mansourkhani S.M., Ardekan A.P., Jouybari L., Daraei N., Peiro K., Mohamadian S., Rezaei M., Heidari M. (2019). Medicinal Plants in the Prevention and Treatment of Colon Cancer. Oxid. Med. Cell. Longev..

[4] Imperatore C., Della Sala G., Casertano M., Luciano P., Aiello A., Laurenzana I., Piccoli C., Menna M. (2019). In Vitro Antiproliferative Evaluation of Synthetic Meroterpenes Inspired by Marine Natural Products. Mar. Drugs.

[5] Manzo E. (2021). Synthesis of Marine Natural Products and Molecules Inspired by Marine Substances. Mar. Drugs.

[6] Karthikeyan C., Moorthy N.S.H.N., Ramasamy S., Vanam U., Manivannan E., Karunagaran D., Trivedi P. (2015). Advances in chalcones with anticancer activities. Recent Pat. Anticancer Drug Discov..

[7] Singh P., Anand A., Kumar V. (2014). Recent developments in biological activities of chalcones: A mini review. Eur. J. Med. Chem..

[8] Zhou B. (2015). Diverse Molecular Targets for Chalcones with Varied Bioactivities. Med. Chem..

[9] Zhuang C., Zhang W., Sheng C., Zhang W., Xing C., Miao Z. (2017). Chalcone: A Privileged Structure in Medicinal Chemistry. Chem. Rev..

[10] Gao F., Huang G., Xiao J. (2020). Chalcone hybrids as potential anticancer agents: Current development, mechanism of action, and structure-activity relationship. Med. Res. Rev..

[11] Mahapatra D.K., Bharti S.K., Asati V. (2017). Chalcone Derivatives: Anti-inflammatory Potential and Molecular Targets Perspectives. Curr. Top. Med. Chem..

[12] Rocha S., Ribeiro D., Fernandes E., Freitas M. (2020). A Systematic Review on Anti-diabetic Properties of Chalcones. Curr. Med. Chem..

[13] Xu S., Chen M., Chen W., Hui J., Ji J., Hu S., Zhou J., Wang Y., Liang G. (2015). Chemopreventive effect of chalcone derivative, L2H17, in colon cancer development. BMC Cancer.

[14] Lin Y., Zhang M., Lu Q., Xie J., Wu J., Chen C. (2019). A novel chalcone derivative exerts anti-inflammatory and anti-oxidant effects after acute lung injury. Aging.

[15] Henry E.J., Bird S.J., Gowland P., Collins M., Cassella J.P. (2020). Ferrocenyl chalcone derivatives as possible antimicrobial agents. J. Antibiot..

[16] de Mello M.V.P., Abrahim-Vieira B.A., Domingos T.F.S., de Jesus J.B., de Sousa A.C.C., Rodrigues C.R., Souza A.M.T. (2018). A comprehensive review of chalcone derivatives as antileishmanial agents. Eur. J. Med. Chem..

[17] Cheng P., Yang L., Huang X., Wang X., Gong M. (2020). Chalcone hybrids and their antimalarial activity. Arch. Pharm..

[18] Gomes M.N., Muratov E.N., Pereira M., Peixoto J.C., Rosseto L.P., Cravo P.V.L., Andrade C.H., Neves B.J. (2017). Chalcone Derivatives: Promising Starting Points for Drug Design. Molecules.

[19] Sahu N.K., Balbhadra S.S., Choudhary J., Kohli D.V. (2012). Exploring pharmacological significance of chalcone scaffold: A review. Curr. Med. Chem..

[20] Bukhari S.N.A., Jasamai M., Jantan I. (2012). Synthesis and biological evaluation of chalcone derivatives (mini review). Mini Rev. Med. Chem..

[21] Zhang E.H., Wang R.F., Guo S.Z., Liu B. (2013). An update on antitumor activity of naturally occurring chalcones. Evid. Based Complement. Alternat. Med..

[22] Rozmer Z., Perjési P. (2014). Naturally occurring chalcones and their biological activities. Phytochem. Rev..

[23] Wang K.L., Yu Y.C., Hsia S.M. (2021). Perspectives on the Role of Isoliquiritigenin in Cancer. Cancers.

[24] Peng F., Tang H., Liu P., Shen J., Guan X., Xie X., Gao J., Xiong L., Jia L., Chen J. (2017). Isoliquiritigenin modulates miR-374a/PTEN/Akt axis to suppress breast cancer tumorigenesis and metastasis. Sci. Rep..

[25] Lin P.-H., Chiang Y.-F., Shieh T.-M., Chen H.-Y., Shih C.-K., Wang T.-H., Wang K.-L., Huang T.-C., Hong Y.-H., Li S.-C. (2020). Dietary Compound Isoliquiritigenin, an Antioxidant from Licorice, Suppresses Triple-Negative Breast Tumor Growth via Apoptotic Death Program Activation in Cell and Xenograft Animal Models. Antioxidants.

[26] Chen H.-Y., Huang T.-C., Shieh T.-M., Wu C.-H., Lin L.-C., Hsia S.-M. (2017). Isoliquiritigenin Induces Autophagy and Inhibits Ovarian Cancer Cell Growth. Int. J. Mol. Sci..

[27] Zhang B., Lai Y., Li Y., Shu N., Wang Z., Wang Y., Li Y., Chen Z. (2018). Antineoplastic activity of isoliquiritigenin, a chalcone compound, in androgen-independent human prostate cancer cells linked to G2/M cell cycle arrest and cell apoptosis. Eur. J. Pharmacol..

[28] Wang C., Chen Y., Wang Y., Liu X., Liu Y., Li Y., Chen H., Fan C., Wu D., Yang J. (2019). Inhibition of COX-2, mPGES-1 and CYP4A by isoliquiritigenin blocks the angiogenic Akt signaling in glioma through ceRNA effect of miR-194-5p and lncRNA NEAT1. J. Exp. Clin. Cancer Res. CR.

[29] Xiang S., Zeng H., Xia F., Ji Q., Xue J., Ren R., Que F., Zhou B. (2021). The dietary flavonoid isoliquiritigenin induced apoptosis and suppressed metastasis in melanoma cells: An in vitro and in vivo study. Life Sci..

[30] Wang N., Wang Z., Peng C., You J., Shen J., Han S., Chen J. (2014). Dietary compound isoliquiritigenin targets GRP78 to chemosensitize breast cancer stem cells via β-catenin/ABCG2 signaling. Carcinogenesis.

[31] Jayasooriya R.G.P.T., Molagoda I.M.N., Park C., Jeong J.-W., Choi Y.H., Moon D.-O., Kim M.-O., Kim G.-Y. (2018). Molecular chemotherapeutic potential of butein: A concise review. Food Chem. Toxicol. Int. J. Publ. Br. Ind. Biol. Res. Assoc..

[32] Padmavathi G., Rathnakaram S.R., Monisha J., Bordoloi D., Roy N.K., Kunnumakkara A.B. (2015). Potential of butein, a tetrahydroxychalcone to obliterate cancer. Phytomedicine.

[33] Di S., Fan C., Ma Z., Li M., Guo K., Han D., Li X., Mu D., Yan X. (2019). PERK/eIF-2α/CHOP Pathway Dependent ROS Generation Mediates Butein-induced Non-small-cell Lung Cancer Apoptosis and G2/M Phase Arrest. Int. J. Biol. Sci..

[34] Zhou Y., Li M., Yu X., Liu T., Li T., Zhou L., Liu W., Li W., Gao F. (2018). Butein suppresses hepatocellular carcinoma growth via modulating Aurora B kinase activity. Int. J. Biol. Sci..

[35] Moon D.-O., Kim M.-O., Choi Y.H., Hyun J.W., Chang W.Y., Kim G.-Y. (2010). Butein induces G(2)/M phase arrest and apoptosis in human hepatoma cancer cells through ROS generation. Cancer Lett..

[36] Moon D.-O., Choi Y.H., Moon S.-K., Kim W.-J., Kim G.-Y. (2010). Butein suppresses the expression of nuclear factor-kappa B-mediated matrix metalloproteinase-9 and vascular endothelial growth factor in prostate cancer cells. Toxicol. In Vitro.

[37] Rasheed Z., Akhtar N., Khan A., Khan K.A., Haqqi T.M. (2010). Butrin, isobutrin, and butein from medicinal plant Butea monosperma selectively inhibit nuclear factor-kappaB in activated human mast cells: Suppression of tumor necrosis factor-alpha, interleukin (IL)-6, and IL-8. J. Pharmacol. Exp. Ther..

[38] Lee D.-S., Jeong G.-S. (2016). Butein provides neuroprotective and anti-neuroinflammatory effects through Nrf2/ARE-dependent haem oxygenase 1 expression by activating the PI3K/Akt pathway. Br. J. Pharmacol..

[39] Sung B., Cho S.-G., Liu M., Aggarwal B.B. (2011). Butein, a tetrahydroxychalcone, suppresses cancer-induced osteoclastogenesis through inhibition of receptor activator of nuclear factor-kappaB ligand signaling. Int. J. Cancer.

[40] Kuete V., Sandjo L.P. (2012). Isobavachalcone: An overview. Chin. J. Integr. Med..

[41] Li B., Xu N., Wan Z., Ma L., Li H., Cai W., Chen X., Huang Z., He Z. (2019). Isobavachalcone exerts anti-proliferative and pro-apoptotic effects on human liver cancer cells by targeting the ERKs/RSK2 signaling pathway. Oncol. Rep..

[42] Li K., Zheng Q., Chen X., Wang Y., Wang D., Wang J. (2018). Isobavachalcone Induces ROS-Mediated Apoptosis Targeting Thioredoxin Reductase 1 in Human Prostate Cancer PC-3 Cells. Oxid. Med. Cell. Longev..

[43] Li Y., Qin X., Li P., Zhang H., Lin T., Miao Z., Ma S. (2019). Isobavachalcone isolated from inhibits cell proliferation and induces apoptosis via inhibiting the AKT/GSK-3β/β-catenin pathway in colorectal cancer cells. Drug Des. Dev. Ther..

[44] Shi J., Chen Y., Chen W., Tang C., Zhang H., Chen Y., Yang X., Xu Z., Wei J., Chen J. (2018). Isobavachalcone sensitizes cells to E2-induced paclitaxel resistance by down-regulating CD44 expression in ER+ breast cancer cells. J. Cell. Mol. Med..

[45] Akihisa T., Tokuda H., Hasegawa D., Ukiya M., Kimura Y., Enjo F., Suzuki T., Nishino H. (2006). Chalcones and other compounds from the exudates of Angelica keiskei and their cancer chemopreventive effects. J. Nat. Prod..

[46] Nishimura R., Tabata K., Arakawa M., Ito Y., Kimura Y., Akihisa T., Nagai H., Sakuma A., Kohno H., Suzuki T. (2007). Isobavachalcone, a chalcone constituent of Angelica keiskei, induces apoptosis in neuroblastoma. Biol. Pharm. Bull..

[47] Pawlak A., Henklewska M., Hernandez Suarez B., Luzny M., Kozlowska E., Obminska-Mrukowicz B., Janeczko T. (2020). Chalcone Methoxy Derivatives Exhibit Antiproliferative and Proapoptotic Activity on Canine Lymphoma and Leukemia Cells. Molecules.

[48] Elkhalifa D., Siddique A.B., Qusa M., Cyprian F.S., El Sayed K., Alali F., Al Moustafa A.E., Khalil A. (2020). Design, synthesis, and validation of novel nitrogen-based chalcone analogs against triple negative breast cancer. Eur. J. Med. Chem..

[49] Kachadourian R., Day B.J., Pugazhenti S., Franklin C.C., Genoux-Bastide E., Mahaffey G., Gauthier C., Di Pietro A., Boumendjel A. (2012). A synthetic chalcone as a potent inducer of glutathione biosynthesis. J. Med. Chem..

[50] Kong Y., Wang K., Edler M.C., Hamel E., Mooberry S.L., Paige M.A., Brown M.L. (2010). A boronic acid chalcone analog of combretastatin A-4 as a potent anti-proliferation agent. Bioorg. Med. Chem..

[51] Schobert R., Biersack B., Dietrich A., Knauer S., Zoldakova M., Fruehauf A., Mueller T. (2009). Pt(II) complexes of a combretastatin A-4 analogous chalcone: Effects of conjugation on cytotoxicity, tumor specificity, and long-term tumor growth suppression. J. Med. Chem..

[52] Yamali C., Gul H.I., Sakagami H., Supuran C.T. (2016). Synthesis and bioactivities of halogen bearing phenolic chalcones and their corresponding bis Mannich bases. J. Enzym. Inhib. Med. Chem..

[53] Zhang S., Li T., Zhang Y., Xu H., Li Y., Zi X., Yu H., Li J., Jin C.Y., Liu H.M. (2016). A new brominated chalcone derivative suppresses the growth of gastric cancer cells in vitro and in vivo involving ROS mediated up-regulation of DR5 and 4 expression and apoptosis. Toxicol. Appl. Pharmacol..

[54] Padhye S., Ahmad A., Oswal N., Dandawate P., Rub R.A., Deshpande J., Swamy K.V., Sarkar F.H. (2010). Fluorinated 2′-hydroxychalcones as garcinol analogs with enhanced antioxidant and anticancer activities. Bioorg. Med. Chem. Lett..

[55] Mahapatra D.K., Bharti S.K., Asati V., Singh S.K. (2019). Perspectives of medicinally privileged chalcone based metal coordination compounds for biomedical applications. Eur. J. Med. Chem..

[56] Singh A.K., Saxena G., Dixit S., Hamidullah, Singh S.K., Singh S.K., Arshad M., Konwar R. (2016). Synthesis, characterization and biological activities of some Ru(II) complexes with substituted chalcones and their applications as chemotherapeutics against breast cancer. J. Mol. Struct..

[57] Jovanovic K.K., Gligorijevic N., Gaur R., Mishra L., Radulovic S. (2016). Anticancer activity of two ruthenium(II)-DMSO-chalcone complexes: Comparison of cytotoxic, pro-apoptotic and antimetastatic potential. J. BUON.

[58] Vutey V., Castelli S., D’Annessa I., Samia L.B., Souza-Fagundes E.M., Beraldo H., Desideri A. (2016). Human topoisomerase IB is a target of a thiosemicarbazone copper(II) complex. Arch. Biochem. Biophys..

[59] Samia L.B., Parrilha G.L., Da Silva J.G., Ramos J.P., Souza-Fagundes E.M., Castelli S., Vutey V., Desideri A., Beraldo H. (2016). Metal complexes of 3-(4-bromophenyl)-1-pyridin-2-ylprop-2-en-1-one thiosemicarbazone: Cytotoxic activity and investigation on the mode of action of the gold(III) complex. Biometals.

[60] Shaveta, Mishra S., Singh P. (2016). Hybrid molecules: The privileged scaffolds for various pharmaceuticals. Eur. J. Med. Chem..

[61] Feng L.-S., Xu Z., Chang L., Li C., Yan X.-F., Gao C., Ding C., Zhao F., Shi F., Wu X. (2020). Hybrid molecules with potential in vitro antiplasmodial and in vivo antimalarial activity against drug-resistant Plasmodium falciparum. Med. Res. Rev..

[62] Meunier B. (2008). Hybrid molecules with a dual mode of action: Dream or reality?. Acc. Chem. Res..

[63] Dai X., Zhang X., Chen W., Chen Y., Zhang Q., Mo S., Lu J. (2021). Dihydroartemisinin: A Potential Natural Anticancer Drug. Int. J. Biol. Sci..

[64] Wong Y.K., Xu C., Kalesh K.A., He Y., Lin Q., Wong W.S.F., Shen H.-M., Wang J. (2017). Artemisinin as an anticancer drug: Recent advances in target profiling and mechanisms of action. Med. Res. Rev..

[65] Fröhlich T., Çapcı Karagöz A., Reiter C., Tsogoeva S.B. (2016). Artemisinin-Derived Dimers: Potent Antimalarial and Anticancer Agents. J. Med. Chem..

[66] Smit F.J., van Biljon R.A., Birkholtz L.M., N’Da D.D. (2015). Synthesis and in vitro biological evaluation of dihydroartemisinyl-chalcone esters. Eur. J. Med. Chem..

[67] Xie L., Zhai X., Ren L., Meng H., Liu C., Zhu W., Zhao Y. (2011). Design, synthesis and antitumor activity of novel artemisinin derivatives using hybrid approach. Chem. Pharm. Bull..

[68] Xie L., Zhai X., Liu C., Li P., Li Y., Guo G., Gong P. (2011). Anti-tumor activity of new artemisinin-chalcone hybrids. Arch. Pharm..

[69] Gaur R., Pathania A.S., Malik F.A., Bhakuni R.S., Verma R.K. (2016). Synthesis of a series of novel dihydroartemisinin monomers and dimers containing chalcone as a linker and their anticancer activity. Eur. J. Med. Chem..

[70] Kapkoti D.S., Singh S., Luqman S., Bhakuni R.S. (2018). Synthesis of novel 1,2,3-triazole based artemisinin derivatives and their antiproliferative activity. New J. Chem..

[71] Ahmad K., Khan M.K.A., Baig M.H., Imran M., Gupta G.K. (2018). Role of Azoles in Cancer Prevention and Treatment: Present and Future Perspectives. Anticancer Agents Med. Chem..

[72] Wang Y., Xue S., Li R., Zheng Z., Yi H., Li Z. (2018). Synthesis and biological evaluation of novel synthetic chalcone derivatives as anti-tumor agents targeting Cat L and Cat K. Bioorg. Med. Chem..

[73] Fathi M.A.A., Abd El-Hafeez A.A., Abdelhamid D., Abbas S.H., Montano M.M., Abdel-Aziz M. (2019). 1,3,4-oxadiazole/chalcone hybrids: Design, synthesis, and inhibition of leukemia cell growth and EGFR, Src, IL-6 and STAT3 activities. Bioorg. Chem..

[74] Hawash M.M.A., Kahraman D.C., Eren F., Cetin Atalay R., Baytas S.N. (2017). Synthesis and biological evaluation of novel pyrazolic chalcone derivatives as novel hepatocellular carcinoma therapeutics. Eur. J. Med. Chem..

[75] ElMonaem H.S.A., Abdel-Aziz N.I., Morsy M.A., Badria F.A., ElSenduny F., El-Ashmawy M.B., Moustafa M.A. (2018). Synthesis, In Vitro Antiproliferative Evaluation and Molecular Docking of New tetrazole-chalcone and tetrazole-pyrazoline Hybrids. J. Appl. Pharm. Sci..

[76] Coman F.-M., Mbaveng A.T., Leonte D., Bencze L.C., Vlase L., Imre S., Kuete V., Efferth T., Zaharia V. (2018). Heterocycles 44. Synthesis, characterization and anticancer activity of new thiazole ortho-hydroxychalcones. Med. Chem. Res..

[77] Hussaini S.M., Yedla P., Babu K.S., Shaik T.B., Chityal G.K., Kamal A. (2016). Synthesis and Biological Evaluation of 1,2,3-triazole tethered Pyrazoline and Chalcone Derivatives. Chem. Biol. Drug. Des..

[78] Ahmed F.F., Abd El-Hafeez A.A., Abbas S.H., Abdelhamid D., Abdel-Aziz M. (2018). New 1,2,4-triazole-Chalcone hybrids induce Caspase-3 dependent apoptosis in A549 human lung adenocarcinoma cells. Eur. J. Med. Chem..

[79] Zhang L., Xu Z. (2019). Coumarin-containing hybrids and their anticancer activities. Eur. J. Med. Chem..

[80] Wang Y., Zhang W., Dong J., Gao J. (2020). Design, synthesis and bioactivity evaluation of coumarin-chalcone hybrids as potential anticancer agents. Bioorg. Chem..

[81] Singh N., Sarkar J., Sashidhara K.V., Ali S., Sinha S. (2014). Anti-tumour activity of a novel coumarin-chalcone hybrid is mediated through intrinsic apoptotic pathway by inducing PUMA and altering Bax/Bcl-2 ratio. Apoptosis.

[82] Ashraf R., Hamidullah, Hasanain M., Pandey P., Maheshwari M., Singh L.R., Siddiqui M.Q., Konwar R., Sashidhara K.V., Sarkar J. (2017). Coumarin-chalcone hybrid instigates DNA damage by minor groove binding and stabilizes p53 through post translational modifications. Sci. Rep..

[83] Sashidhara K.V., Kumar A., Kumar M., Sarkar J., Sinha S. (2010). Synthesis and in vitro evaluation of novel coumarin-chalcone hybrids as potential anticancer agents. Bioorg. Med. Chem. Lett..

[84] Elshemy H.A.H., Zaki M.A. (2017). Design and synthesis of new coumarin hybrids and insight into their mode of antiproliferative action. Bioorg. Med. Chem..

[85] Patel K., Karthikeyan C., Solomon V.R., Moorthy N.S.H.N., Lee H., Sahu K., Deora G.S., Trivedi P. (2011). Synthesis of Some Coumarinyl Chalcones and their Antiproliferative Activity Against Breast Cancer Cell Lines. Lett. Drug Des. Discov..

[86] Suwito H., Hardiyanti H., Ul Haq K., Kristanti A., Khasanah M. (2018). (E)-3-[3-(4-Morpholinophenyl)acryloyl]-2H-chromen-2-one. Molbank.

[87] Dadashpour S., Emami S. (2018). Indole in the target-based design of anticancer agents: A versatile scaffold with diverse mechanisms. Eur. J. Med. Chem..

[88] Kumari A., Singh R.K. (2019). Medicinal chemistry of indole derivatives: Current to future therapeutic prospectives. Bioorg. Chem..

[89] Patil S.A., Patil R., Miller D.D. (2012). Indole molecules as inhibitors of tubulin polymerization: Potential new anticancer agents. Future Med. Chem..

[90] Wan Y., Li Y., Yan C., Yan M., Tang Z. (2019). Indole: A privileged scaffold for the design of anti-cancer agents. Eur. J. Med. Chem..

[91] Mirzaei H., Shokrzadeh M., Modanloo M., Ziar A., Riazi G.H., Emami S. (2017). New indole-based chalconoids as tubulin-targeting antiproliferative agents. Bioorg. Chem..

[92] Wang G., Li C., He L., Lei K., Wang F., Pu Y., Yang Z., Cao D., Ma L., Chen J. (2014). Design, synthesis and biological evaluation of a series of pyrano chalcone derivatives containing indole moiety as novel anti-tubulin agents. Bioorg. Med. Chem..

[93] Yan J., Chen J., Zhang S., Hu J., Huang L., Li X. (2016). Synthesis, Evaluation, and Mechanism Study of Novel Indole-Chalcone Derivatives Exerting Effective Antitumor Activity Through Microtubule Destabilization in Vitro and in Vivo. J. Med. Chem..

[94] Cong H., Zhao X., Castle B.T., Pomeroy E.J., Zhou B., Lee J., Wang Y., Bian T., Miao Z., Zhang W. (2018). An Indole-Chalcone Inhibits Multidrug-Resistant Cancer Cell Growth by Targeting Microtubules. Mol. Pharm..

[95] Smit F.J., Bezuidenhout J.J., Bezuidenhout C.C., N’Da D.D. (2016). Synthesis and in vitro biological activities of ferrocenyl–chalcone amides. Med. Chem. Res..

[96] Pérès B., Nasr R., Zarioh M., Lecerf-Schmidt F., Di Pietro A., Baubichon-Cortay H., Boumendjel A. (2017). Ferrocene-embedded flavonoids targeting the Achilles heel of multidrug-resistant cancer cells through collateral sensitivity. Eur. J. Med. Chem..

[97] Farzaneh S., Zeinalzadeh E., Daraei B., Shahhosseini S., Zarghi A. (2018). New Ferrocene Compounds as Selective Cyclooxygenase (COX-2) Inhibitors: Design, Synthesis, Cytotoxicity and Enzyme-inhibitory Activity. Anti-cancer agents Med. Chem..

[98] Mphahlele M.J., Maluleka M.M., Parbhoo N., Malindisa S.T. (2018). Synthesis, Evaluation for Cytotoxicity and Molecular Docking Studies of Benzo[c]furan-Chalcones for Potential to Inhibit Tubulin Polymerization and/or EGFR-Tyrosine Kinase Phosphorylation. Int. J. Mol. Sci..

[99] Silbermann K., Shah C.P., Sahu N.U., Juvale K., Stefan S.M., Kharkar P.S., Wiese M. (2019). Novel chalcone and flavone derivatives as selective and dual inhibitors of the transport proteins ABCB1 and ABCG2. Eur. J. Med. Chem..

[100] Jeon K.-H., Yu H.-B., Kwak S.Y., Kwon Y., Na Y. (2016). Synthesis and topoisomerases inhibitory activity of heteroaromatic chalcones. Bioorg. Med. Chem..

[101] Bandeira P.N., Lemos T.L., Santos H.S., de Carvalho M.C., Pinheiro D.P., de Moraes Filho M.O., Pessoa C., Barros-Nepomuceno F.W., Rodrigues T.H., Ribeiro P.R. (2019). Synthesis, structural characterization, and cytotoxic evaluation of chalcone derivatives. Med. Chem. Res..

[102] Bagul C., Rao G.K., Makani V.K.K., Tamboli J.R., Pal-Bhadra M., Kamal A. (2017). Synthesis and biological evaluation of chalcone-linked pyrazolo[1,5-]pyrimidines as potential anticancer agents. Medchemcomm.

[103] Abou-Zied H.A., Youssif B.G.M., Mohamed M.F.A., Hayallah A.M., Abdel-Aziz M. (2019). EGFR inhibitors and apoptotic inducers: Design, synthesis, anticancer activity and docking studies of novel xanthine derivatives carrying chalcone moiety as hybrid molecules. Bioorg. Chem..

[104] Gibson M.Z., Nguyen M.A., Zingales S.K. (2018). Design, Synthesis, and Evaluation of (2-(Pyridinyl)methylene)-1-tetralone Chalcones for Anticancer and Antimicrobial Activity. Med. Chem..

[105] Wang M., Xu S., Wu C., Liu X., Tao H., Huang Y., Liu Y., Zheng P., Zhu W. (2016). Design, synthesis and activity of novel sorafenib analogues bearing chalcone unit. Bioorg. Med. Chem. Lett..

[106] Xu F., Li W., Shuai W., Yang L., Bi Y., Ma C., Yao H., Xu S., Zhu Z., Xu J. (2019). Design, synthesis and biological evaluation of pyridine-chalcone derivatives as novel microtubule-destabilizing agents. Eur. J. Med. Chem..

[107] Li W., Xu F., Shuai W., Sun H., Yao H., Ma C., Xu S., Yao H., Zhu Z., Yang D.-H. (2019). Discovery of Novel Quinoline-Chalcone Derivatives as Potent Antitumor Agents with Microtubule Polymerization Inhibitory Activity. J. Med. Chem..

[108] Tabbi A., Tebbani D., Caporale A., Saturnino C., Nabavi S.F., Giuseppe P., Arra C., Canturk Z., Turan-Zitouni G., Merazig H. (2017). New Adamantyl Chalcones: Synthesis, Antimicrobial and Anticancer Activities. Curr. Top. Med. Chem..

[109] Peña-Solórzano D., Scholler M., Bernhardt G., Buschauer A., König B., Ochoa-Puentes C. (2018). Tariquidar-Related Chalcones and Ketones as ABCG2 Modulators. ACS Med. Chem. Lett..

[110] Tseng C.-H., Tzeng C.-C., Hsu C.-Y., Cheng C.-M., Yang C.-N., Chen Y.-L. (2015). Discovery of 3-phenylquinolinylchalcone derivatives as potent and selective anticancer agents against breast cancers. Eur. J. Med. Chem..

[111] Lindamulage I.K., Vu H.-Y., Karthikeyan C., Knockleby J., Lee Y.-F., Trivedi P., Lee H. (2017). Novel quinolone chalcones targeting colchicine-binding pocket kill multidrug-resistant cancer cells by inhibiting tubulin activity and MRP1 function. Sci. Rep..

[112] Wani Z.A., Guru S.K., Rao A.V.S., Sharma S., Mahajan G., Behl A., Kumar A., Sharma P.R., Kamal A., Bhushan S. (2016). A novel quinazolinone chalcone derivative induces mitochondrial dependent apoptosis and inhibits PI3K/Akt/mTOR signaling pathway in human colon cancer HCT-116 cells. Food Chem. Toxicol. Int. J. Publ. Br. Ind. Biol. Res. Assoc..

[113] Han X., Peng B., Xiao B.-B., Sheng-Li C., Yang C.-R., Wang W.-Z., Wang F.-C., Li H.-Y., Yuan X.-L., Shi R. (2019). Synthesis and evaluation of chalcone analogues containing a 4-oxoquinazolin-2-yl group as potential anti-tumor agents. Eur. J. Med. Chem..

[114] Kraege S., Stefan K., Juvale K., Ross T., Willmes T., Wiese M. (2016). The combination of quinazoline and chalcone moieties leads to novel potent heterodimeric modulators of breast cancer resistance protein (BCRP/ABCG2). Eur. J. Med. Chem..

[115] Marković V., Debeljak N., Stanojković T., Kolundžija B., Sladić D., Vujčić M., Janović B., Tanić N., Perović M., Tešić V. (2015). Anthraquinone-chalcone hybrids: Synthesis, preliminary antiproliferative evaluation and DNA-interaction studies. Eur. J. Med. Chem..

[116] Qiu H.-Y., Wang F., Wang X., Sun W.-X., Qi J.-L., Pang Y.-J., Yang R.-W., Lu G.-H., Wang X.-M., Yang Y.-H. (2017). Design, Synthesis, and Biological Evaluation of Chalcone-Containing Shikonin Derivatives as Inhibitors of Tubulin Polymerization. ChemMedChem.

[117] Guimarães D.G., de Assis Gonsalves A., Rolim L.A., Araújo E.C., Dos Anjos Santos V.L., Silva M.F.S., de Cássia Evangelista de Oliveira F., da Costa M.P., Pessoa C., Fonseca Goulart M.O. (2020). Naphthoquinone-based hydrazone hybrids: Synthesis and potent activity against cancer cell lines. Med. Chem..

[118] Ng H.-L., Chen S., Chew E.-H., Chui W.-K. (2016). Applying the designed multiple ligands approach to inhibit dihydrofolate reductase and thioredoxin reductase for anti-proliferative activity. Eur. J. Med. Chem..

[119] Ng H.-L., Ma X., Chew E.-H., Chui W.-K. (2017). Design, Synthesis, and Biological Evaluation of Coupled Bioactive Scaffolds as Potential Anticancer Agents for Dual Targeting of Dihydrofolate Reductase and Thioredoxin Reductase. J. Med. Chem..

[120] Fu D.-J., Li J.-H., Yang J.-J., Li P., Zhang Y.-B., Liu S., Li Z.-R., Zhang S.-Y. (2019). Discovery of novel chalcone-dithiocarbamates as ROS-mediated apoptosis inducers by inhibiting catalase. Bioorg. Chem..

[121] Fu D.-J., Zhang S.-Y., Liu Y.-C., Zhang L., Liu J.-J., Song J., Zhao R.-H., Li F., Sun H.-H., Liu H.-M. (2016). Design, synthesis and antiproliferative activity studies of novel dithiocarbamate-chalcone derivates. Bioorg. Med. Chem. Lett..

[122] Batovska D.I., Todorova I.T. (2010). Trends in utilization of the pharmacological potential of chalcones. Curr Clin Pharmacol.

[123] Sharma R., Kumar R., Kodwani R., Kapoor S., Khare A., Bansal R., Khurana S., Singh S., Thomas J., Roy B. (2015). A Review on Mechanisms of Anti Tumor Activity of Chalcones. Anticancer Agents Med. Chem..

[124] Mahapatra D.K., Bharti S.K., Asati V. (2015). Anti-cancer chalcones: Structural and molecular target perspectives. Eur. J. Med. Chem..

[125] Stein Y., Rotter V., Aloni-Grinstein R. (2019). Gain-of-Function Mutant p53: All the Roads Lead to Tumorigenesis. Int. J. Mol. Sci..

[126] Wu D., Prives C. (2018). Relevance of the p53-MDM2 axis to aging. Cell Death Differ..

[127] Karni-Schmidt O., Lokshin M., Prives C. (2016). The Roles of MDM2 and MDMX in Cancer. Annu. Rev. Pathol..

[128] Silva G., Marins M., Fachin A.L., Lee S.-H., Baek S.J. (2016). Anti-cancer activity of trans-chalcone in osteosarcoma: Involvement of Sp1 and p53. Mol. Carcinog..

[129] Dos Santos M.B., Bertholin Anselmo D., de Oliveira J.G., Jardim-Perassi B.V., Alves Monteiro D., Silva G., Gomes E., Lucia Fachin A., Marins M., de Campos Zuccari D.A.P. (2019). Antiproliferative activity and p53 upregulation effects of chalcones on human breast cancer cells. J. Enzym. Inhib. Med. Chem..

[130] Silva G., Marins M., Chaichanasak N., Yoon Y., Fachin A.L., Pinhanelli V.C., Regasini L.O., Dos Santos M.B., Ayusso G.M., Marques B.C. (2018). Trans-chalcone increases p53 activity via DNAJB1/HSP40 induction and CRM1 inhibition. PLoS ONE.

[131] Siqueira E.D.S., Concato V.M., Tomiotto-Pellissier F., Silva T.F., Bortoleti B., Goncalves M.D., Costa I.N., Junior W.A.V., Pavanelli W.R., Panis C. (2021). Trans-chalcone induces death by autophagy mediated by p53 up-regulation and beta-catenin down-regulation on human hepatocellular carcinoma HuH7.5 cell line. Phytomedicine.

[132] Seba V., Silva G., Santos M.B.D., Baek S.J., Franca S.C., Fachin A.L., Regasini L.O., Marins M. (2018). Chalcone Derivatives 4’-Amino-1-Naphthyl-Chalcone (D14) and 4’-Amino-4-Methyl-1-Naphthyl-Chalcone (D15) Suppress Migration and Invasion of Osteosarcoma Cells Mediated by p53 Regulating EMT-Related Genes. Int. J. Mol. Sci..

[133] Cabral B.L.S., da Silva A.C.G., de Avila R.I., Cortez A.P., Luzin R.M., Liao L.M., de Souza Gil E., Sanz G., Vaz B.G., Sabino J.R. (2017). A novel chalcone derivative, LQFM064, induces breast cancer cells death via p53, p21, KIT and PDGFRA. Eur. J. Pharm. Sci..

[134] Peyrot V., Leynadier D., Sarrazin M., Briand C., Rodriquez A., Nieto J.M., Andreu J.M. (1989). Interaction of tubulin and cellular microtubules with the new antitumor drug MDL 27048. A powerful and reversible microtubule inhibitor. J. Biol. Chem..

[135] Ducki S., Rennison D., Woo M., Kendall A., Chabert J.F., McGown A.T., Lawrence N.J. (2009). Combretastatin-like chalcones as inhibitors of microtubule polymerization. Part 1: Synthesis and biological evaluation of antivascular activity. Bioorg. Med. Chem..

[136] Ducki S., Mackenzie G., Greedy B., Armitage S., Chabert J.F., Bennett E., Nettles J., Snyder J.P., Lawrence N.J. (2009). Combretastatin-like chalcones as inhibitors of microtubule polymerisation. Part 2: Structure-based discovery of alpha-aryl chalcones. Bioorg. Med. Chem..

[137] Kamal A., Kumar G.B., Vishnuvardhan M.V., Shaik A.B., Reddy V.S., Mahesh R., Sayeeda I.B., Kapure J.S. (2015). Synthesis of phenstatin/isocombretastatin-chalcone conjugates as potent tubulin polymerization inhibitors and mitochondrial apoptotic inducers. Org. Biomol. Chem..

[138] Kode J., Kovvuri J., Nagaraju B., Jadhav S., Barkume M., Sen S., Kasinathan N.K., Chaudhari P., Mohanty B.S., Gour J. (2020). Synthesis, biological evaluation, and molecular docking analysis of phenstatin based indole linked chalcones as anticancer agents and tubulin polymerization inhibitors. Bioorg. Chem..

[139] Yan W., Xiangyu C., Ya L., Yu W., Feng X. (2019). An orally antitumor chalcone hybrid inhibited HepG2 cells growth and migration as the tubulin binding agent. Invest. New Drugs.

[140] Canela M.-D., Noppen S., Bueno O., Prota A.E., Bargsten K., Sáez-Calvo G., Jimeno M.-L., Benkheil M., Ribatti D., Velázquez S. (2017). Antivascular and antitumor properties of the tubulin-binding chalcone TUB091. Oncotarget.

[141] Alswah M., Bayoumi A.H., Elgamal K., Elmorsy A., Ihmaid S., Ahmed H.E.A. (2017). Design, Synthesis and Cytotoxic Evaluation of Novel Chalcone Derivatives Bearing Triazolo[4,3-a]-quinoxaline Moieties as Potent Anticancer Agents with Dual EGFR Kinase and Tubulin Polymerization Inhibitory Effects. Molecules.

[142] Hoesel B., Schmid J.A. (2013). The complexity of NF-κB signaling in inflammation and cancer. Mol. Cancer.

[143] Perkins N.D. (2012). The diverse and complex roles of NF-κB subunits in cancer. Nat. Rev. Cancer.

[144] Gamble C., McIntosh K., Scott R., Ho K.H., Plevin R., Paul A. (2012). Inhibitory kappa B Kinases as targets for pharmacological regulation. Br. J. Pharmacol..

[145] Baldwin A.S. (2012). Regulation of cell death and autophagy by IKK and NF-κB: Critical mechanisms in immune function and cancer. Immunol. Rev..

[146] Yadav V.R., Prasad S., Sung B., Aggarwal B.B. (2011). The role of chalcones in suppression of NF-kappaB-mediated inflammation and cancer. Int. Immunopharmacol..

[147] Pandey M.K., Sandur S.K., Sung B., Sethi G., Kunnumakkara A.B., Aggarwal B.B. (2007). Butein, a tetrahydroxychalcone, inhibits nuclear factor (NF)-kappaB and NF-kappaB-regulated gene expression through direct inhibition of IkappaBalpha kinase beta on cysteine 179 residue. J. Biol. Chem..

[148] Lee Y.H., Jeon S.H., Kim S.H., Kim C., Lee S.J., Koh D., Lim Y., Ha K., Shin S.Y. (2012). A new synthetic chalcone derivative, 2-hydroxy-3’,5,5’-trimethoxychalcone (DK-139), suppresses the Toll-like receptor 4-mediated inflammatory response through inhibition of the Akt/NF-kappaB pathway in BV2 microglial cells. Exp. Mol. Med..

[149] Kim J.-Y., Park S.J., Yun K.-J., Cho Y.-W., Park H.-J., Lee K.-T. (2008). Isoliquiritigenin isolated from the roots of Glycyrrhiza uralensis inhibits LPS-induced iNOS and COX-2 expression via the attenuation of NF-kappaB in RAW 264.7 macrophages. Eur. J. Pharmacol..

[150] Zeng J., Chen Y., Ding R., Feng L., Fu Z., Yang S., Deng X., Xie Z., Zheng S. (2017). Isoliquiritigenin alleviates early brain injury after experimental intracerebral hemorrhage via suppressing ROS- and/or NF-κB-mediated NLRP3 inflammasome activation by promoting Nrf2 antioxidant pathway. J. Neuroinflamm..

[151] Liao Y., Tan R.-Z., Li J.-C., Liu T.-T., Zhong X., Yan Y., Yang J.-K., Lin X., Fan J.-M., Wang L. (2020). Isoliquiritigenin Attenuates UUO-Induced Renal Inflammation and Fibrosis by Inhibiting Mincle/Syk/NF-Kappa B Signaling Pathway. Drug Des. Dev. Ther..

[152] Folmer F., Blasius R., Morceau F., Tabudravu J., Dicato M., Jaspars M., Diederich M. (2006). Inhibition of TNFalpha-induced activation of nuclear factor kappaB by kava (Piper methysticum) derivatives. Biochem. Pharmacol..

[153] Lv H., Yang H., Wang Z., Feng H., Deng X., Cheng G., Ci X. (2019). Nrf2 signaling and autophagy are complementary in protecting lipopolysaccharide/d-galactosamine-induced acute liver injury by licochalcone A. Cell Death Dis..

[154] Ruibin J., Bo J., Danying W., Jianguo F., Linhui G. (2020). Cardamonin induces G2/M phase arrest and apoptosis through inhibition of NF-κB and mTOR pathways in ovarian cancer. Aging.

[155] Badroon N.A., Abdul Majid N., Alshawsh M.A. (2020). Antiproliferative and Apoptotic Effects of Cardamonin against Hepatocellular Carcinoma HepG2 Cells. Nutrients.

[156] Hou S., Yuan Q., Yu N., Liu B., Huang G., Yuan X. (2019). Cardamonin attenuates chronic inflammation and tumorigenesis in colon. Cell Cycle.

[157] Harikumar K.B., Kunnumakkara A.B., Ahn K.S., Anand P., Krishnan S., Guha S., Aggarwal B.B. (2009). Modification of the cysteine residues in IkappaBalpha kinase and NF-kappaB (p65) by xanthohumol leads to suppression of NF-kappaB-regulated gene products and potentiation of apoptosis in leukemia cells. Blood.

[158] Venkateswararao E., Sharma V.K., Yun J., Kim Y., Jung S.H. (2014). Anti-proliferative effect of chalcone derivatives through inactivation of NF-kappaB in human cancer cells. Bioorg. Med. Chem..

[159] Gan F.F., Zhang R., Ng H.L., Karuppasamy M., Seah W., Yeap W.H., Ong S.M., Hadadi E., Wong S.C., Chui W.K. (2018). Novel dual-targeting anti-proliferative dihydrotriazine-chalcone derivatives display suppression of cancer cell invasion and inflammation by inhibiting the NF-kappaB signaling pathway. Food Chem. Toxicol..

[160] Mirossay L., Varinska L., Mojzis J. (2017). Antiangiogenic Effect of Flavonoids and Chalcones: An Update. Int. J. Mol. Sci..

[161] Cerezo A.B., Winterbone M.S., Moyle C.W.A., Needs P.W., Kroon P.A. (2015). Molecular structure-function relationship of dietary polyphenols for inhibiting VEGF-induced VEGFR-2 activity. Mol. Nutr. Food Res..

[162] Shanmugam M.K., Warrier S., Kumar A.P., Sethi G., Arfuso F. (2017). Potential Role of Natural Compounds as Anti-Angiogenic Agents in Cancer. Curr. Vasc. Pharmacol..

[163] Ma Y., Xu B., Yu J., Huang L., Zeng X., Shen X., Ren C., Ben-David Y., Luo H. (2020). Fli-1 Activation through Targeted Promoter Activity Regulation Using a Novel 3′, 5′-diprenylated Chalcone Inhibits Growth and Metastasis of Prostate Cancer Cells. Int. J. Mol. Sci..

[164] Iheagwam F.N., Ogunlana O.O., Ogunlana O.E., Isewon I., Oyelade J. (2019). Potential Anti-Cancer Flavonoids Isolated From Caesalpinia bonduc Young Twigs and Leaves: Molecular Docking and In Silico Studies. Bioinform. Biol. Insights.

[165] Wang Z., Wang N., Han S., Wang D., Mo S., Yu L., Huang H., Tsui K., Shen J., Chen J. (2013). Dietary compound isoliquiritigenin inhibits breast cancer neoangiogenesis via VEGF/VEGFR-2 signaling pathway. PLoS ONE.

[166] Kwon S.J., Park S.Y., Kwon G.T., Lee K.W., Kang Y.H., Choi M.S., Yun J.W., Jeon J.H., Jun J.G., Park J.H. (2013). Licochalcone E present in licorice suppresses lung metastasis in the 4T1 mammary orthotopic cancer model. Cancer Prev. Res..

[167] Saito K., Matsuo Y., Imafuji H., Okubo T., Maeda Y., Sato T., Shamoto T., Tsuboi K., Morimoto M., Takahashi H. (2018). Xanthohumol inhibits angiogenesis by suppressing nuclear factor-kappaB activation in pancreatic cancer. Cancer Sci..

[168] Wang L., Chen G., Lu X., Wang S., Han S., Li Y., Ping G., Jiang X., Li H., Yang J. (2015). Novel chalcone derivatives as hypoxia-inducible factor (HIF)-1 inhibitor: Synthesis, anti-invasive and anti-angiogenic properties. Eur. J. Med. Chem..

[169] Wang C., Li Y., Chen H., Zhang J., Zhang J., Qin T., Duan C., Chen X., Liu Y., Zhou X. (2017). Inhibition of CYP4A by a novel flavonoid FLA-16 prolongs survival and normalizes tumor vasculature in glioma. Cancer Lett..

[170] Jeong J.H., Jang H.J., Kwak S., Sung G.J., Park S.H., Song J.H., Kim H., Na Y., Choi K.C. (2019). Novel TGF-beta1 inhibitor antagonizes TGF-beta1-induced epithelial-mesenchymal transition in human A549 lung cancer cells. J. Cell. Biochem..

[171] Stanojkovic T., Markovic V., Matic I.Z., Mladenovic M.P., Petrovic N., Krivokuca A., Petkovic M., Joksovic M.D. (2018). Highly selective anthraquinone-chalcone hybrids as potential antileukemia agents. Bioorg. Med. Chem. Lett..

[172] Robey R.W., Pluchino K.M., Hall M.D., Fojo A.T., Bates S.E., Gottesman M.M. (2018). Revisiting the role of ABC transporters in multidrug-resistant cancer. Nat. Rev. Cancer.

[173] Vasan N., Baselga J., Hyman D.M. (2019). A view on drug resistance in cancer. Nature.

[174] Bois F., Boumendjel A., Mariotte A.M., Conseil G., Di Petro A. (1999). Synthesis and biological activity of 4-alkoxy chalcones: Potential hydrophobic modulators of P-glycoprotein-mediated multidrug resistance. Bioorg. Med. Chem..

[175] Bois F., Beney C., Boumendjel A., Mariotte A.M., Conseil G., Di Pietro A. (1998). Halogenated chalcones with high-affinity binding to P-glycoprotein: Potential modulators of multidrug resistance. J. Med. Chem..

[176] Parveen Z., Brunhofer G., Jabeen I., Erker T., Chiba P., Ecker G.F. (2014). Synthesis, biological evaluation and 3D-QSAR studies of new chalcone derivatives as inhibitors of human P-glycoprotein. Bioorg. Med. Chem..

[177] Ngo T.D., Tran T.D., Le M.T., Thai K.M. (2016). Computational predictive models for P-glycoprotein inhibition of in-house chalcone derivatives and drug-bank compounds. Mol. Divers..

[178] Gu X., Ren Z., Tang X., Peng H., Ma Y., Lai Y., Peng S., Zhang Y. (2012). Synthesis and biological evaluation of bifendate-chalcone hybrids as a new class of potential P-glycoprotein inhibitors. Bioorg. Med. Chem..

[179] Yin H., Dong J., Cai Y., Shi X., Wang H., Liu G., Tang Y., Liu J., Ma L. (2019). Design, synthesis and biological evaluation of chalcones as reversers of P-glycoprotein-mediated multidrug resistance. Eur. J. Med. Chem..

[180] Li J., Zheng L., Yan M., Wu J., Liu Y., Tian X., Jiang W., Zhang L., Wang R. (2020). Activity and mechanism of flavokawain A in inhibiting P-glycoprotein expression in paclitaxel resistance of lung cancer. Oncol. Lett..

[181] Li J., Zheng L., Wang R., Sun D., Liang S., Wu J., Liu Y., Tian X., Li T., Yang Y. (2020). Synergistic Combination of Sodium Aescinate-Stabilized, Polymer-Free, Twin-Like Nanoparticles to Reverse Paclitaxel Resistance. Int. J. Nanomed..

[182] Wu C.P., Lusvarghi S., Hsiao S.H., Liu T.C., Li Y.Q., Huang Y.H., Hung T.H., Ambudkar S.V. (2020). Licochalcone A Selectively Resensitizes ABCG2-Overexpressing Multidrug-Resistant Cancer Cells to Chemotherapeutic Drugs. J. Nat. Prod..

[183] Winter E., Devantier Neuenfeldt P., Chiaradia-Delatorre L.D., Gauthier C., Yunes R.A., Nunes R.J., Creczynski-Pasa T.B., Di Pietro A. (2014). Symmetric bis-chalcones as a new type of breast cancer resistance protein inhibitors with a mechanism different from that of chromones. J. Med. Chem..

[184] Boumendjel A., McLeer-Florin A., Champelovier P., Allegro D., Muhammad D., Souard F., Derouazi M., Peyrot V., Toussaint B., Boutonnat J. (2009). A novel chalcone derivative which acts as a microtubule depolymerising agent and an inhibitor of P-gp and BCRP in in-vitro and in-vivo glioblastoma models. BMC Cancer.

[185] Han Y., Riwanto M., Go M.L., Ee P.L. (2008). Modulation of breast cancer resistance protein (BCRP/ABCG2) by non-basic chalcone analogues. Eur. J. Pharm. Sci..

[186] Cai C.Y., Zhang W., Wang J.Q., Lei Z.N., Zhang Y.K., Wang Y.J., Gupta P., Tan C.P., Wang B., Chen Z.S. (2020). Biological evaluation of non-basic chalcone CYB-2 as a dual ABCG2/ABCB1 inhibitor. Biochem. Pharmacol..

[187] Shimiz K., Kondo R., Sakai K., Buabarn S., Dilokkunanant U. (2000). A geranylated chalcone with 5alpha-reductase inhibitory properties from Artocarpus incisus. Phytochemistry.

[188] Le Bail J.C., Pouget C., Fagnere C., Basly J.P., Chulia A.J., Habrioux G. (2001). Chalcones are potent inhibitors of aromatase and 17beta-hydroxysteroid dehydrogenase activities. Life Sci..

[189] Mourad A.A.E., Mourad M.A.E., Jones P.G. (2020). Novel HDAC/Tubulin Dual Inhibitor: Design, Synthesis and Docking Studies of α-Phthalimido-Chalcone Hybrids as Potential Anticancer Agents with Apoptosis-Inducing Activity. Drug Des. Dev. Ther..

[190] Mohamed M.F.A., Shaykoon M.S.A., Abdelrahman M.H., Elsadek B.E.M., Aboraia A.S., Abuo-Rahma G.E.-D.A.A. (2017). Design, synthesis, docking studies and biological evaluation of novel chalcone derivatives as potential histone deacetylase inhibitors. Bioorg. Chem..

[191] Nedungadi D., Binoy A., Pandurangan N., Nair B.G., Mishra N. (2021). Proteasomal dysfunction and ER stress triggers 2’-hydroxy-retrochalcone-induced paraptosis in cancer cells. Cell Biol. Int..

[192] Li X., Pham V., Tippin M., Fu D., Rendon R., Song L., Uchio E., Hoang B.H., Zi X. (2019). Flavokawain B targets protein neddylation for enhancing the anti-prostate cancer effect of Bortezomib via Skp2 degradation. Cell Commun. Signal..

[193] Jobst B., Weigl J., Michl C., Vivarelli F., Pinz S., Amslinger S., Rascle A. (2016). Inhibition of interleukin-3- and interferon- α-induced JAK/STAT signaling by the synthetic α-X-2’,3,4,4’-tetramethoxychalcones α-Br-TMC and α-CF3-TMC. Biol. Chem..

[194] Yuan X., Li T., Xiao E., Zhao H., Li Y., Fu S., Gan L., Wang Z., Zheng Q., Wang Z. (2014). Licochalcone B inhibits growth of bladder cancer cells by arresting cell cycle progression and inducing apoptosis. Food Chem. Toxicol. Int. J. Publ. Br. Ind. Biol. Res. Assoc..

[195] Sun X., Zhang J., Wang Z., Liu B., Zhu S., Zhu L., Peng B. (2019). Licorice isoliquiritigenin-encapsulated mesoporous silica nanoparticles for osteoclast inhibition and bone loss prevention. Theranostics.

[196] Zhou W., Zhang W., Peng Y., Jiang Z.-H., Zhang L., Du Z. (2020). Design, Synthesis and Anti-Tumor Activity of Novel Benzimidazole-Chalcone Hybrids as Non-Intercalative Topoisomerase II Catalytic Inhibitors. Molecules.

[197] Li P.-H., Jiang H., Zhang W.-J., Li Y.-L., Zhao M.-C., Zhou W., Zhang L.-Y., Tang Y.-D., Dong C.-Z., Huang Z.-S. (2018). Synthesis of carbazole derivatives containing chalcone analogs as non-intercalative topoisomerase II catalytic inhibitors and apoptosis inducers. Eur. J. Med. Chem..

[198] Wang T.-T., Chen Z.-Z., Xie P., Zhang W.-J., Du M.-Y., Liu Y.-T., Zhu H.-Y., Guo Y.-S. (2019). Isoliquiritigenin suppresses the proliferation and induced apoptosis via miR-32/LATS2/Wnt in nasopharyngeal carcinoma. Eur. J. Pharmacol..

[199] Predes D., Oliveira L.F.S., Ferreira L.S.S., Maia L.A., Delou J.M.A., Faletti A., Oliveira I., Amado N.G., Reis A.H., Fraga C.A.M. (2019). The Chalcone Lonchocarpin Inhibits Wnt/β-Catenin Signaling and Suppresses Colorectal Cancer Proliferation. Cancers.

[200] Wang L.H., Li H.H., Li M., Wang S., Jiang X.R., Li Y., Ping G.F., Cao Q., Liu X., Fang W.H. (2015). SL4, a chalcone-based compound, induces apoptosis in human cancer cells by activation of the ROS/MAPK signalling pathway. Cell Prolif..

[201] Lee J.A., Kim D.J., Hwang O. (2019). KMS99220 Exerts Anti-Inflammatory Effects, Activates the Nrf2 Signaling and Interferes with IKK, JNK and p38 MAPK via HO-1. Mol. Cells.

[202] Hseu Y.-C., Lee M.-S., Wu C.-R., Cho H.-J., Lin K.-Y., Lai G.-H., Wang S.-Y., Kuo Y.-H., Kumar K.J.S., Yang H.-L. (2012). The chalcone flavokawain B induces G2/M cell-cycle arrest and apoptosis in human oral carcinoma HSC-3 cells through the intracellular ROS generation and downregulation of the Akt/p38 MAPK signaling pathway. J. Agric. Food Chem..

[203] Wang J., Qi Q., Zhou W., Feng Z., Huang B., Chen A., Zhang D., Li W., Zhang Q., Jiang Z. (2018). Inhibition of glioma growth by flavokawain B is mediated through endoplasmic reticulum stress induced autophagy. Autophagy.

[204] Jin J., Qiu S., Wang P., Liang X., Huang F., Wu H., Zhang B., Zhang W., Tian X., Xu R. (2019). Cardamonin inhibits breast cancer growth by repressing HIF-1α-dependent metabolic reprogramming. J. Exp. Clin. Cancer Res. CR.

[205] Ziegler S., Pries V., Hedberg C., Waldmann H. (2013). Target identification for small bioactive molecules: Finding the needle in the haystack. Angew. Chem. Int. Ed. Engl..

[206] Marquina S., Maldonado-Santiago M., Sanchez-Carranza J.N., Antunez-Mojica M., Gonzalez-Maya L., Razo-Hernandez R.S., Alvarez L. (2019). Design, synthesis and QSAR study of 2′-hydroxy-4′-alkoxy chalcone derivatives that exert cytotoxic activity by the mitochondrial apoptotic pathway. Bioorg. Med. Chem..

[207] Song Y.Q., Guan X.Q., Weng Z.M., Liu J.L., Chen J., Wang L., Cui L.T., Fang S.Q., Hou J., Ge G.B. (2021). Discovery of hCES2A inhibitors from Glycyrrhiza inflata via combination of docking-based virtual screening and fluorescence-based inhibition assays. Food Funct..

[208] George R.F., Kandeel M., El-Ansary D.Y., El Kerdawy A.M. (2020). Some 1,3,5-trisubstituted pyrazoline derivatives targeting breast cancer: Design, synthesis, cytotoxic activity, EGFR inhibition and molecular docking. Bioorg. Chem..

[209] Lee Y., Kim B.S., Ahn S., Koh D., Lee Y.H., Shin S.Y., Lim Y. (2016). Anticancer and structure-activity relationship evaluation of 3-(naphthalen-2-yl)-N,5-diphenyl-pyrazoline-1-carbothioamide analogs of chalcone. Bioorg. Chem..

[210] Shin S.Y., Yoon H., Ahn S., Kim D.-W., Kim S.H., Koh D., Lee Y.H., Lim Y. (2013). Chromenylchalcones showing cytotoxicity on human colon cancer cell lines and in silico docking with aurora kinases. Bioorg. Med. Chem..

[211] Bisht K., Wagner K.H., Bulmer A.C. (2010). Curcumin, resveratrol and flavonoids as anti-inflammatory, cyto- and DNA-protective dietary compounds. Toxicology.

[212] Kommidi D.R., Pagadala R., Rana S., Singh P., Shintre S.A., Koorbanally N.A., Jonnalagadda S.B., Moodley B. (2015). Novel carbapenem chalcone derivatives: Synthesis, cytotoxicity and molecular docking studies. Org. Biomol. Chem..

[213] Muchtaridi M., Syahidah H.N., Subarnas A., Yusuf M., Bryant S.D., Langer T. (2017). Molecular Docking and 3D-Pharmacophore Modeling to Study the Interactions of Chalcone Derivatives with Estrogen Receptor Alpha. Pharmaceuticals.

[214] Riswanto F.D.O., Rawa M.S.A., Murugaiyah V., Salin N.H., Istyastono E.P., Hariono M., Wahab H.A. (2019). Anti-Cholinesterase Activity of Chalcone Derivatives: Synthesis, In Vitro Assay and Molecular Docking Study. Med. Chem..

[215] Narayanapillai S.C., Leitzman P., O’Sullivan M.G., Xing C. (2014). Flavokawains a and B in kava, not dihydromethysticin, potentiate acetaminophen-induced hepatotoxicity in C57BL/6 mice. Chem. Res. Toxicol..

[216] Tugcu G., Kirmizibekmez H., Aydin A. (2020). The integrated use of in silico methods for the hepatotoxicity potential of Piper methysticum. Food Chem. Toxicol..

[217] Qian Y., Yang Y., Wang K., Zhou W., Dang Y., Zhu M., Li F., Ji G. (2019). 2’-Hydroxychalcone Induced Cytotoxicity via Oxidative Stress in the Lipid-Loaded Hepg2 Cells. Front. Pharmacol..

[218] Jeong C.H., Park H.B., Jang W.J., Jung S.H., Seo Y.H. (2014). Discovery of hybrid Hsp90 inhibitors and their anti-neoplastic effects against gefitinib-resistant non-small cell lung cancer (NSCLC). Bioorg. Med. Chem. Lett..

[219] Oh Y.J., Seo Y.H. (2017). A novel chalcone-based molecule, BDP inhibits MDAMB231 triple-negative breast cancer cell growth by suppressing Hsp90 function. Oncol. Rep..

[220] Schopf F.H., Biebl M.M., Buchner J. (2017). The HSP90 chaperone machinery. Nat. Rev. Mol. Cell Biol..

[221] Butler L.M., Ferraldeschi R., Armstrong H.K., Centenera M.M., Workman P. (2015). Maximizing the Therapeutic Potential of HSP90 Inhibitors. Mol. Cancer Res..

[222] Salehi B., Quispe C., Chamkhi I., El Omari N., Balahbib A., Sharifi-Rad J., Bouyahya A., Akram M., Iqbal M., Docea A.O. (2020). Pharmacological Properties of Chalcones: A Review of Preclinical Including Molecular Mechanisms and Clinical Evidence. Front. Pharmacol..

[223] Haefner B. (2003). Drugs from the deep: Marine natural products as drug candidates. Drug Discov. Today.

[224] Blunt J.W., Copp B.R., Munro M.H.G., Northcote P.T., Prinsep M.R. (2005). Marine natural products. Nat. Prod. Rep..

[225] De Luca D., Lauritano C. (2020). In Silico Identification of Type III PKS Chalcone and Stilbene Synthase Homologs in Marine Photosynthetic Organisms. Biology.

